# Molecular and Heterojunction Device Engineering of Solution‐Processed Conjugated Reticular Oligomers: Enhanced Photoelectrochemical Hydrogen Evolution through High‐Effective Exciton Separation

**DOI:** 10.1002/advs.202308535

**Published:** 2024-03-07

**Authors:** Boying Zhang, Huimin Gao, Yazhou Kang, Xiaoming Li, Qing Li, Pengda Zhai, Diane Hildebrandt, Xinying Liu, Yue Wang, Shanlin Qiao

**Affiliations:** ^1^ College of Chemistry and Pharmaceutical Engineering Hebei University of Science and Technology Shijiazhuang 050018 China; ^2^ Department of Chemical Engineering Faculty of Engineering and the Built Environment University of Johannesburg Doornfontein 2028 South Africa; ^3^ Department of Chemical and Biochemical Engineering Rutgers University Piscataway New Jersey 08854 USA; ^4^ Institute for Catalysis and Energy Solutions University of South Africa Florida 1709 South Africa

**Keywords:** bulk heterojunction devices, conjugated reticular oligomers, covalent organic frameworks, photoelectrochemical hydrogen evolution, solution processability photoelectrode

## Abstract

Covalent organic frameworks (COFs) face limited processability challenges as photoelectrodes in photoelectrochemical water reduction. Herein, sub‐10 nm benzothiazole‐based colloidal conjugated reticular oligomers (CROs) are synthesized using an aqueous nanoreactor approach, and the end‐capping molecular strategy to engineer electron‐deficient units onto the periphery of a CRO nanocrystalline lattices (named CROs‐Cg). This results in stable and processable “electronic inks” for flexible photoelectrodes. CRO‐BtzTp‐Cg and CRO‐TtzTp‐Cg expand the absorption spectrum into the infrared region and improve fluorescence lifetimes. Heterojunction device engineering is used to develop interlayer heterojunction and bulk heterojunction (BHJ) photoelectrodes with a hole transport layer, electron transport layer, and the main active layers, using a CROs/CROs‐Cg or one‐dimensional (1D) electron‐donating polymer HP18 mixed solution via spinning coating. The ITO/CuI/CRO‐TtzTp‐Cg‐HP18/SnO_2_/Pt photoelectrode shows a photocurrent of 94.9 µA cm^‒2^ at 0.4 V versus reversible hydrogen electrode (RHE), which is 47.5 times higher than that of ITO/Bulk‐TtzTp. Density functional theory calculations show reduced energy barriers for generating adsorbed H* intermediates and increased electron affinity in CROs‐Cg. Mott‐Schottky and charge density difference analyses indicate enhanced charge carrier densities and accelerated charge transfer kinetics in BHJ devices. This study lays the groundwork for large‐scale production of COF nanomembranes and heterojunction structures, offering the potential for cost‐effective, printable energy systems.

## Graphical abstract

Sub‐10 nm colloidal conjugated reticular oligomers (CROs) are synthesized using an aqueous nanoreactor. An end‐capping molecular strategy is employed to introduce electron‐deficient units onto the periphery of a two‐dimensional CROs nanocrystalline lattice, denoted as CROs‐Cg. This process yields stable, solution‐processable ″electronic inks,″ facilitating the fabrication of flexible photoelectrodes for photoelectrochemical water reduction through device engineering, resulting in outstanding photocurrent performance.

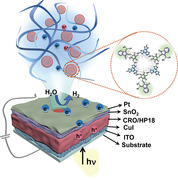

## 1 Introduction

Photoelectrochemical (PEC) water splitting has garnered increasing attention as a promising approach for harnessing solar energy to produce clean, renewable hydrogen fuel. It is a green energy technology that has been researched extensively, as it addresses certain challenges in terms of energy and the environment.^[^ ^1^, ^2^ ^]^ PEC water splitting combines the benefits of photocatalysis and electrocatalysis, with light energy serving as an additional input that enhances catalytic efficiency significantly.^[^ ^3^, ^4^ ^]^ The production of photogenerated carriers is coupled with electron conduction through a photoelectron pathway, which accelerates electron and ion transfer and promotes the reaction kinetics of PEC water splitting.^[^ ^5^, ^6^ ^]^

The core component of the panel‐type photoelectrode used is the semiconductive active layer. It combines light absorption and electron transport in an integrated system,^[^ ^7^ ^]^ as well as provides a large reaction interface and many active sites, which facilitates contact between reactant and catalyst, so enhancing the reaction rate.^[^ ^8^ ^]^ However, there are still significant challenges with the panel‐type photoelectrode in practical applications, i.e., i) *Semiconductor design*. The performance of PEC devices significantly depends not only on the light absorption capability and the efficiency of photogenerated charge separation of the photoelectrode semiconductor but also on the efficiency and kinetic characteristics of the electrocatalyst.^[^ ^9^ ^]^ In electrocatalysts, achieving fast electron and proton transfer is one of the keys to improving PEC performance since it is closely related to photogenerated charge separation. In addition, most photocatalytic semiconductors have a wide band gap that limits their ability to absorb photons in the short wavelength range.^[^ ^10^ ^]^ The photogenerated charge separation efficiency may be affected by surface defects on the panel‐type photoelectrode, which reduces photoelectric conversion efficiency.^[^ ^11^ ^]^ ii) *Film preparation technology*. Effective control of the uniformity and quality of the panel‐type photoelectrode film is crucial for PEC devices, as heterogeneous deposition and defects can impair device performance significantly. iii) *Economic feasibility*. The cost of equipment and processes must be considered during the film preparation process, especially when aiming to meet high efficiency and large‐scale production requirements. This constrains the commercialization of film PEC technology.

Organic semiconductors offer the advantage of customizability through precise adjustments to their molecular structure and chemical composition.^[^ ^12^ ^]^ Furthermore, organic semiconductor films are remarkably flexible and can attach seamlessly to curved or irregular substrates, which makes them suitable for low‐cost, high‐throughput fabrication of large‐area optoelectronic devices in PEC applications. Two‐dimensional covalent organic frameworks (2D‐COFs) are a rapidly developing type of crystalline porous polymer that have highly ordered structures, permanent porosity, and functional diversity.^[^ ^13^, ^14^, ^15^ ^]^ The active sites, the position of the frontier orbitals, and energy bandgaps can be easily customized by organic synthesis using readily available building blocks, various linkages, and diverse topologies.^[^ ^16^, ^17^, ^18^, ^19^, ^20^, ^21^ ^]^ These characteristics, along with the extended intralayer π conjugation and columnar π arrays, endow COFs with a potent charge transfer ability for PEC water splitting applications.^[^ ^22^, ^23^ ^]^ However, despite the potential for aligned one‐dimensional (1D) open channels, which can enhance charge carrier transport and mass transfer, the application of COFs in optoelectronic devices is inferior to small conjugated molecular and linear polymer catalysts.^[^ ^24^ ^]^ The current bottleneck in advancing PEC water splitting lies in the limited preparation method and processability of COFs, which are typically obtained as insoluble solids. As a result, there is a dearth of efficient solutions for fabricating COF films or heterojunction structures that facilitate efficient charge harvesting in COFs.^[^ ^25^, ^26^, ^27^ ^]^ Research has been explored on electrophoretic deposition,^[^ ^27^ ^]^ substrate orientation,^[^ ^25^ ^]^ and solvothermal synthesis^[^ ^26^ ^]^ when preparing COF films as photoelectrodes used in PEC water splitting. However, challenges persist in terms of controlling the thickness of the film and achieving high photoelectric conversion efficiency. Hence, there is a need to devise a novel strategy for preparing solution‐processed COF photoelectrode films to achieve efficient exciton separation and promote PEC water splitting. Although the top‐down approach for exfoliating bulk 2D COF powders into nanoparticles has been thoroughly investigated,^[^ ^28^, ^29^, ^30^ ^]^ it is difficult to produce stable dispersed COF solutions with a definite concentration and uniform size. This results in challenges in meeting the requirements of smooth and consecutive active layers in optoelectronic device fabrication. The bottom‐up growth approach for colloidal COFs is a novel strategy that allows for precise adjustment of reaction conditions to avoid crystal precipitation. Some research groups have reported developing colloidal COFs based on boronate ester, imine, and *β*‐keto‐enamine linkages.^[^ ^31^, ^32^, ^33^, ^34^ ^]^ However, the reported particle size of colloidal COFs currently ranges from a few hundred nanometers to micrometers, which prevents the preparation of uniform, smooth film with a thickness of several hundred nanometers via flexible printing. Our group used a catanionic micellar size‐confined nanoreactor to synthesize three distinct stabilized colloidal solutions of well‐defined conjugated reticular oligomers (CROs), which can be considered as either defect‐free COF segments or soluble conjugated nanocrystals.^[^ ^35^ ^]^ The minimum particle size of the three CROs was ≈10 nm, which may be more favorable for producing consecutive, homogeneous films compared to bulk COFs with insoluble solids.

Given the above, our investigations focused on synthesizing sub‐10 nm solution‐processed CRO nanocrystals, which are akin to electronic ink, to enable large‐scale inkjet printing for fabricating the panel‐type photoelectrode. A benzothiazole subunit was used as the photoactive center. The aromatic benzothiazole molecules exhibited an exceptional ability for light absorption, allowing for effective absorption of both visible and near‐ultraviolet radiation.^[^ ^36^, ^37^ ^]^ Moreover, from an electronic structure perspective, the band structure of benzothiazole molecules facilitated efficient separation and transmission of photoexcited electrons and holes. The energy band level position allows for sufficient absorption of energy upon photoexcitation, transitioning electrons from the valence to the conduction band (CB) and enabling them to participate in photochemical reactions.

**Scheme 1:**
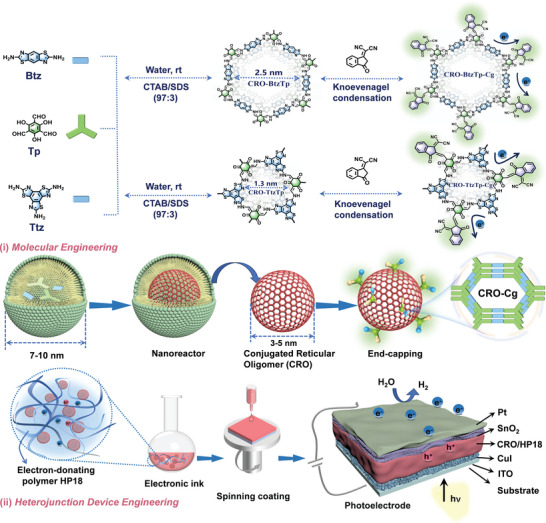
Molecular and heterojunction device engineering of colloidal CROs for PEC water splitting.

For fine molecular structural control, we employed the end‐capping strategy to engineer electron‐deficient units onto the periphery of the CROs lattice by molecular engineering, named CROs‐Cg. This allowed us to reinforce the optoelectronic characteristics. In comparison to Bulk‐BtzTp and Bulk‐TtzTp, small molecule CROs and CROs‐Cg exhibit superior capability in producing a smooth and continuously active layer for PEC applications. By novel engineering of heterojunction devices, we have revolutionized the design of an interlayer heterojunction device (IHJ) and bulk heterojunction (BHJ) device. The process involves optimizing a hole‐transport layer (HTL), an electron transport layer (ETL), an active layer comprising CROs or CRO‐Cg, and an electron‐donating polymer (for BHJ). A schematic of the process is shown in **Scheme** **1**. Through the synergy of molecular and device engineering, we successfully improved the light‐harvesting efficiency and alleviated the charge recombination in photoelectrodes, which resulted in a notable enhancement of the photocurrent density. ITO/CuI/CRO‐BtzTp‐HP18/SnO_2_/Pt, ITO/CuI/CRO‐BtzTp‐Cg‐HP18/SnO_2_/Pt, ITO/CuI/CRO‐TtzTp‐HP18/SnO_2_/Pt and ITO/CuI/CRO‐TtzTp‐Cg‐HP18/SnO_2_/Pt showed a current difference (Δ*J*) of 28.9, 60.6, 43.7 and 94.9 µA cm^‒2^ at 0.4 V (versus reversible hydrogen electrode, RHE), respectively. This is 10.0, 16.8, 13.2, and 20.2 times higher than that of ITO/CRO‐BtzTp, ITO/CRO‐BtzTp‐Cg, ITO/CRO‐TtzTp and ITO/CRO‐TtzTp‐Cg. This study establishes the fundamental groundwork for the large‐scale fabrication of solution‐processed COF nanomembranes and heterojunction photoelectrodes, showcasing immense potential in the realm of producing flexible solar energy conversion devices.

## 2 Results and Discussion

### 2.1 Synthesis Design and Characterization of CROs and CROs‐Cg

Two stabilized colloidal solutions of CROs below 10 nm were synthesized by co‐polycondensation of *bis*‐benzothiazole diamine (Btz) and *tris*‐benzothiazole triamine (Ttz) with triformylphloroglucinol (Tp) in the catanionic micellar system.^[^ ^35^, ^38^ ^]^ Building blocks containing *bis*‐ and *tris*‐benzothiazole moieties are expected to exert a strong influence, owing to their heightened affinity for H* and their semiconducting properties, which enhance charge carrier mobility.^[^ ^39^ ^]^

The CROs synthesized with a symmetrical combination of *C*_3_+*C*_2_ and *C*_3_+*C*_3_ were designated CRO‐BtzTp and CRO‐TtzTp, respectively. The micellar solution consisted of anionic sodium dodecyl sulfate (SDS) and cationic hexadecyltrimethylammonium bromide (CTAB) surfactants, which formed a synergistic A+B system. Molecular engineering was then employed to incorporate the electron‐deficient dicyanomethyleneindianone (2HIC) moiety as a capping group (Cg) on the periphery of CRO‐BtzTp and CRO‐TtzTp via Knoevenagel Condensation. The push‐pull effect can be introduced in CRO‐BtzTp‐Cg and CRO‐TtzTp‐Cg by covalently bonding the electron‐withdrawing 2HIC, thus enhancing π‒π packing and resonance effects to improve the capacity for light absorption. 2HIC can modulate the electron distribution and energy level of the whole CRO lattices, which facilitates charge separation. Bulk‐BtzTp, Bulk‐TtzTp, Bulk‐BtzTp‐Cg, and Bulk‐TtzTp‐Cg were synthesized for comparative purposes. (Please refer to the Supporting Information section for further details).

**Figure 1:**
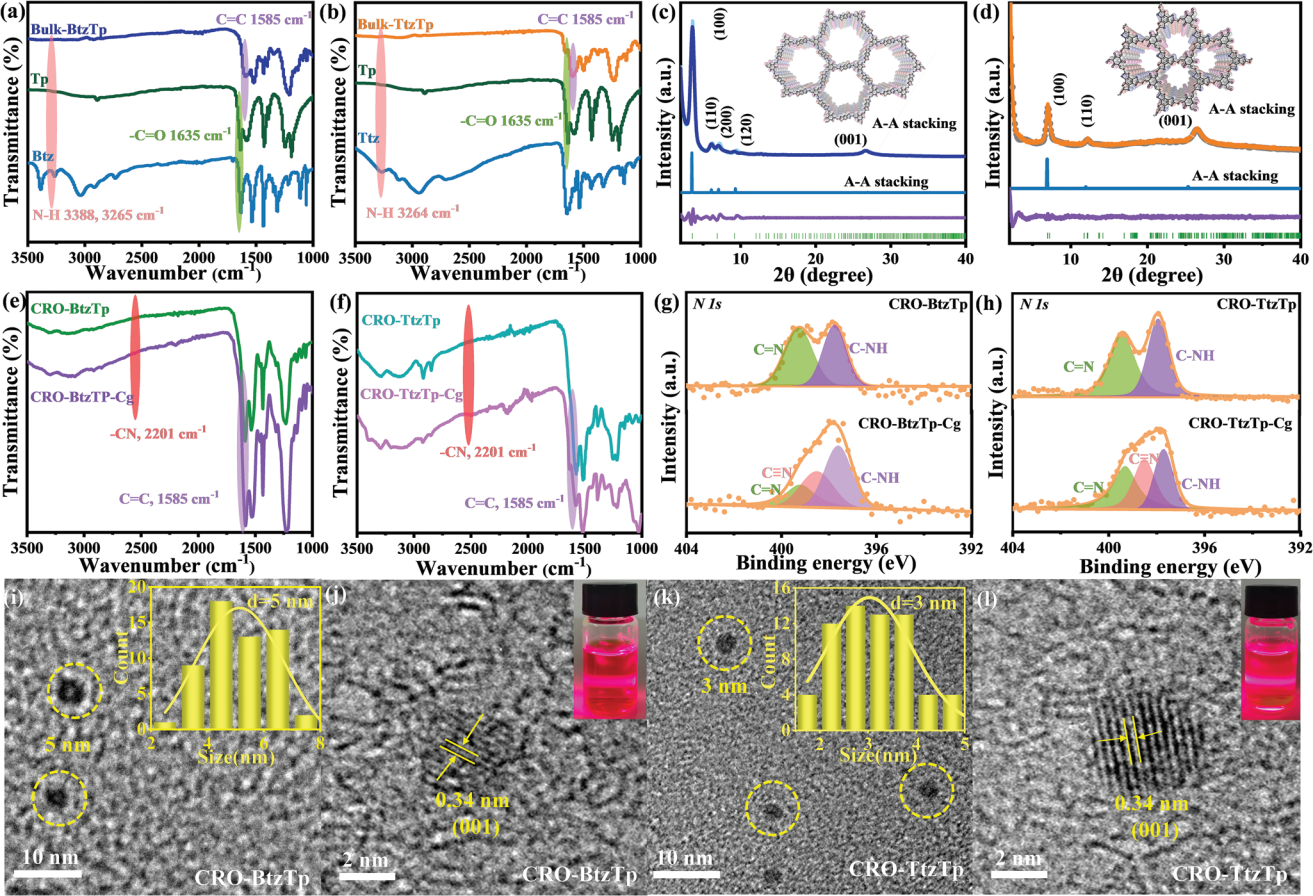
FT‐IR spectra of a) Bulk‐BtzTp; b) Bulk‐TtzTp. Experimental, refined, simulated P‐XRD patterns and the refinement differences of c) Bulk‐BtzTp; d) Bulk‐TtzTp. FT‒IR spectra of e) CRO‐BtzTp and CRO‐BtzTp‐Cg; f) CRO‐TtzTp and CRO‐TtzTp‐Cg. Deconvoluted N 1*s* of g) CRO‐BtzTp and CRO‐BtzTp‐Cg; h) CRO‐TtzTp and CRO‐TtzTp‐Cg. HR‐TEM images of i,j) CRO‐BtzTp; k,l) CRO‐TtzTp. The insets are photographs of a transparent, homogeneous colloidal solution of CRO‐BtzTp and CRO‐TtzTp that show the Willis−Tyndall scattering behavior following irradiation in (j) and (l) and the size distributions obtained in (i) and (k).

Fourier transform infrared (FT‐IR) spectroscopy was conducted to confirm the formation of the designed *β‐ketamine‐linked* Bulk‐BtzTp and Bulk‐TtzTp. **Figure** **1**a,b shows the characteristic C═C and C═O stretching vibration bands of Bulk‐BtzTp and Bulk‐TtzTp at 1590 and 1635 cm^‒1^, respectively, and the disappearance of the N─H stretching vibration band signal at 3325 and 3324 cm^‒1^. The presence of ─CN stretching vibration bands at 2201 cm^‒1^ in the FT‐IR spectra of Bulk‐BtzTp‐Cg and Bulk‐TtzTp‐Cg (Figure S1, Supporting Information) indicates the successful integration of the 2HIC groups and indicates the applicability of this method.

The crystalline structure of the two proposed COFs, Bulk‐BtzTp and Bulk‐TtzTp, was verified utilizing powder X‐ray diffraction (P‐XRD). Bulk‐BtzTp exhibited intense diffraction peaks at 3.58°, 6.22°, 7.17°, 9.35°, and 26.66°, which correspond to the (100), (110), (200), (120), and (001) crystal facets, respectively (Figure 1c). Bulk‐TtzTp displays diffraction peaks at 6.99°, 12.22°, and 26.51°, which are assigned to the (100), (110) and (001) crystallographic planes, respectively (Figure 1d).

To perform theoretical calculations on P‐XRD patterns, we assembled a 2D hexagonal layered structure to construct a unit cell structural model for Bulk‐BtzTp and Bulk‐TtzTp (Figure 1c,d, inset). The simulation eclipsed (AA staking) models exhibit excellent concordance with the experimental P‐XRD results for the two COFs (Figure 1c,d). Moreover, Pawley refinements of experimental P‐XRD patterns regarding AA staking models were utilized for Bulk‐BtzTp (Figure 1c) and Bulk‐TtzTp (Figure 1d), which confirmed the accuracy of peak assignment with minimal deviation. The P‐XRD patterns of Bulk‐BtzTp‐Cg and Bulk‐TtzTp‐Cg are the same as that of Bulk‐BtzTp and Bulk‐TtzTp. This suggests that implementing the end‐capping strategy does not induce any discernible disruption to the underlying crystal structures (Figure S2, Supporting Information). The high‐resolution transmission electron microscope (HR‐TEM) images of Bulk‐BtzTp and Bulk‐TtzTp reveal lattice fringes measuring 2.35 and 1.26 nm, respectively, which correspond to the (100) facets of the hexagonal lattice (Figure S3, Supporting Information). This agrees with the experimental and simulated P‐XRD results of Bulk‐BtzTp and Bulk‐TtzTp, which suggests that both compounds have an exceptional crystal quality and exhibit a well‐organized AA stacked structure.

The FT‐IR spectra of CRO‐BtzTp and CRO‐TtzTp are shown in Figure 1e,f, respectively. They exhibit the characteristic stretching vibration bands of C═C at 1585 cm^‒1^, which indicates successful polycondensation between Tp and Btz/Ttz. The ─CN stretching vibration bands at 2201 cm^‒1^ in the FT‐IR spectra of CRO‐BtzTp‐Cg and CRO‐TtzTp‐Cg are evidence of successful integration of the 2HIC groups in the micellar system.

X‐ray photoelectron spectrometer (XPS) detects the presence of carbon, nitrogen, sulfur, and oxygen in CRO‐BtzTp, CRO‐TtzTp, CRO‐BtzTp‐Cg, and CRO‐TtzTp‐Cg (Figures S4‒S6, Supporting Information). This conclusion was further corroborated by TEM‐EDS analysis (Figures S7 and S8, Supporting Information). Elemental mapping images revealed a uniform distribution of carbon, nitrogen, sulfur, and oxygen in CRO‐BtzTp, CRO‐TtzTp, CRO‐BtzTp‐Cg, and CRO‐TtzTp‐Cg. The deconvoluted N 1*s* spectra of CRO‐BtzTp‐Cg and CRO‐TtzTp‐Cg exhibit three distinct peaks at 397.60, 398.52, and 399.20 eV, which correspond to the chemical groups C─NH, ─CN, and C═N. In contrast, only the C═N and C─NH groups are detected in the XPS spectra of CRO‐BtzTp and CRO‐TtzTp (Figure 1g,h). This shows that 2HIC was covalently connected on the CRO‐BtzTp and CRO‐TtzTp.

The micromorphology of the CRO‐BtzTp and CRO‐TtzTp were characterized using HR‐TEM. As shown in Figure 1i,k, both CRO‐BtzTp and CRO‐TtzTp exhibited excellent dispersion and homogeneity and displayed a relatively uniform size distribution with an average diameter of ≈5 and 3 nm, respectively. The CRO solution exhibited a distinct Willis‐Tyndall scattering pattern after being stored at room temperature for 30 days, which is evidence of stabilized colloidal solutions of CRO‐BtzTp and CRO‐TtzTp. The HR‐TEM images of CRO‐BtzTp and CRO‐TtzTp reveal a lattice fringe with 0.33 nm spacing, which corresponds to the (001) facet (Figure 1j,l) and which indicates that both compounds are reticular nanocrystals. We also have conducted experiments to determine the particle size of synthesized CRO‐BtzTp, CRO‐BtzTp‐Cg, CRO‐TtzTp, and CRO‐TtzTp‐Cg using dynamic light scattering (DLS) analysis (Figure S9, Supporting Information). They exhibit unimodal particle size distributions, measuring 4.3, 5.1, 2.8, and 3.8 nm, respectively.

The average diameter was obtained from TEM, and we propose that the possible structures of CRO‐BtzTp and CRO‐TtzTp are shown in Figures S10a and S11a, Supporting Information, respectively. CRO‐BtzTp and CRO‐TtzTp possess a significant number of terminal residual aldehyde groups, that enable end capping with 2HIC via the Knoevenagel Condensation method (Figures S10b and S11b, Supporting Information). The molecular weight of single‐layer CRO‐BtzTp and CRO‐TtzTp was determined to be about 6800 and 3700, respectively, using *ChemDraw* software.

It is worth noting that Bulk‐BtzTp, CRO‐BtzTp, CRO‐BtzTp‐Cg, Bulk‐TtzTp, CRO‐TtzTp, and CRO‐TtzTp‐Cg were determined to have small contact angles, i.e., 15°, 46°, 28°, 17°, 22°, and 42° (Figure S12, Supporting Information). The heightened structural polarity and augmented water affinity are primarily attributed to the integration of heteroatom‐rich Btz and Ttz units. This results in enhanced surface wettability, which is a key attribute for aqueous PEC.

**Figure 2:**
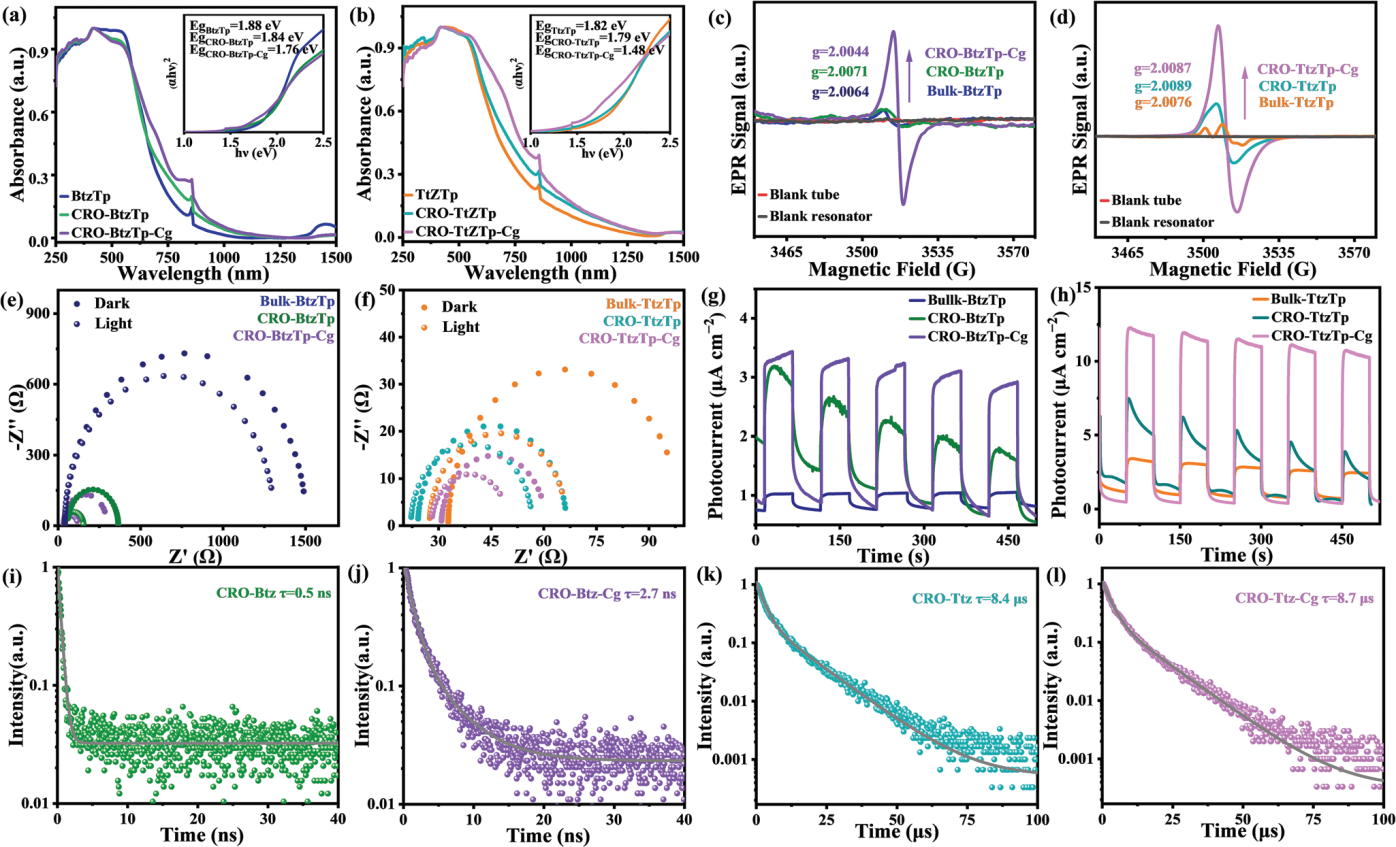
Solid state UV–vis diffuse reflectance spectra of a) BtzTp, CRO‐BtzTp, and CRO‐BtzTp‐Cg; b) TtzTp, CRO‐TtzTp, and CRO‐TtzTp‐Cg. Insets (a) and (b) are optical band gap calculations from the *Tauc* plots of BtzTp, CRO‐BtzTp, CRO‐BtzTp‐Cg, TtzTp, CRO‐TtzTp and CRO‐TtzTp‐Cg. EPR spectra of c) BtzTp, CRO‐BtzTp, and CRO‐BtzTp‐Cg; d) TtzTp, CRO‐TtzTp, and CRO‐TtzTp‐Cg; and blank tube as well as blank resonator following light irradiation. Nyquist plots of e) BtzTp, CRO‐BtzTp, and CRO‐BtzTp‐Cg; f) TtzTp, CRO‐TtzTp, and CRO‐TtzTp‐Cg. Transient photocurrent measurement of g) BtzTp, CRO‐BtzTp, and CRO‐BtzTp‐Cg; h) TtzTp, CRO‐TtzTp, and CRO‐TtzTp‐Cg at 0 V under chopped AM 1.5 illumination. Time‐resolved PL decay profiles of i) CRO‐BtzTp; j) CRO‐BtzTp‐Cg; k) CRO‐TtzTp; l) CRO‐TtzTp‐Cg.

Ultraviolet‐visible diffuse reflectance spectra (UV–vis DRS) of both Bulk‐BtzTp and CRO‐BtzTp exhibit broad absorption bands from the ultraviolet to near‐infrared regions, with the maximum absorption peak at 650 and 670 nm (**Figure** **2**a,b). The red shift in the absorption spectrum of CRO‐BtzTp toward longer wavelengths may be attributed to surface effects and terminal carbonyl groups, as well as *J‐type* aggregation with chromophores (benzothiazole units) between adjacent molecules, which leads to changes in the band structure and electronic state.^[^ ^40^, ^41^, ^42^ ^]^

After 2HIC is incorporated into CRO‐BtzTp, the absorption boundary of CRO‐BtzTp‐Cg is further extended up to about 1000 nm. The maximum absorption peak observed at 710 nm is red‐shifted by 40 nm compared to that of CRO‐BtzTp. A similar trend was observed in the TtzTp‐based catalyst but with a greater degree of variation. The CRO‐TtzTp‐Cg displays a characteristic maximum absorption band at 830 nm and exhibits a significant red shift of 130 nm with a long‐tail absorption band extending to 1250 nm, compared to that of CRO‐TtzTp. The band gap energy of Bulk‐BtzTp, CRO‐BtzTp, CRO‐BtzTp‐Cg, Bulk‐TtzTp, CRO‐TtzTp, and CRO‐TtzTp‐Cg was estimated using *Tauc* plots and found to be 1.88, 1.84, 1.76, 1.82, 1.79, and 1.48 eV, respectively. The electron‐withdrawing 2HIC introduces the electron push‐pull effect into the π‐conjugated CROs,^[^ ^11^, ^43^ ^]^ which narrows the band gap and excites the electron to the Lowest Unoccupied Molecular Orbital (LUMO) energy level. The optimized energy band structure further enhances light absorption and electron collection on active sites.

The carrier generation and transport functions of Bulk‐BtzTp, CRO‐BtzTp, CRO‐BtzTp‐Cg, Bulk‐TtzTp, CRO‐TtzTp, and CRO‐TtzTp‐Cg were examined. Following light irradiation, electron paramagnetic resonance (EPR) spectra detected a signal at *g* = 2.0044−2.0089. This indicates the presence of unpaired electrons in the CB of COFs and confirms the generation of photo‐induced carriers through photoexcitation^[^ ^44^ ^]^ (Figure 2c,d). The EPR spectra reveal that the signal intensity of CRO‐BtzTp‐Cg is 11 and 10 times greater than that of Bulk‐BtzTp and CRO‐BtzTp, respectively. Similarly, the signal intensity of CRO‐TtzTp‐Cg is observed to be 8 and 4 times higher than that of Bulk‐TtzTp and CRO‐TtzTp. It is indicated that the presence of a more concentrated unpaired electron population than that of Bulk‐BtzTp, CRO‐BtzTp, Bulk‐TtzTp, and CRO‐TtzTp.^[^ ^45^ ^]^ In addition, Bulk‐BtzTp, with its lowest *g‐factor* (2.0044), exhibits high structural regularity and symmetry, leading to a homogeneous electronic spin environment with minimal spin‐orbit coupling. Conversely, CRO‐BtzTp and CRO‐BtzTp‐Cg show higher *g* values of 2.0071 and 2.0064 due to surface effects, quantum confinement, and uneven electron spin distribution. The introduction of an electron‐withdrawing group in CRO‐BtzTp‐Cg further reduces spin density. Increased *g* values in Bulk‐TtzTp, CRO‐TtzTp, and CRO‐TtzTp‐Cg result from smaller pore sizes, enhancing electron localization, and strong donor‐acceptor interactions between Ttz and Tp, causing a reorganization of electron density and spin distribution.

Electrochemical impedance spectroscopy (EIS) was used to confirm carrier transport.^[^ ^46^, ^47^ ^]^ In the Nyquist plots illustrated (Figure 2e,f), it is evident that the charge‐transfer resistance (*R*_ct_) exhibited a reduction for bulk COFs, CROs, and CROs‐Cg upon exposure to visible light. In the Bode plots (Figure S14, Supporting Information), it is noticeable that under illumination conditions, the impedance magnitude for bulk COFs, CROs, and CROs‐Cg decreases, accompanied by a shift in the peak of the phase angle toward higher frequencies. This phenomenon is commonly interpreted as a decrease in *R*_ct_, aligning with the observed reduction in the diameter of the semicircles in the Nyquist plots. It is suggested that photo‐induced charge carriers were generated under light irradiation.^[^ ^48^ ^]^ Notably, CRO‐BtzTp‐Cg and CRO‐TtzTp‐Cg show further enhanced carrier mobility, as confirmed by the reduced resistance observed in the Nyquist plots (Table S1, Supporting Information). Figure 2e,f shows the transient photocurrent measurements that confirm that both CRO‐BtzTp‐Cg and CRO‐TtzTp‐Cg exhibit a rapid photo‐response to irradiation, generating a significant photocurrent density of ≈3 and 12 µA cm^−2^, respectively. This surpasses that observed for Bulk‐BtzTp, CRO‐BtzTp, Bulk‐TtzTp, and CRO‐TtzTp.^[^ ^49^, ^50^ ^]^

In order to gain deeper insight into the photophysical characteristics of the photoinduced charge carriers, photoluminescence (PL) measurement was performed using both steady‐state and time‐resolved methodologies. In steady‐state PL spectra (Figure S15, Supporting Information), the observed PL quenching in CRO‐BtzTp‐Cg and CRO‐TtzTp‐Cg provides evidence of a decrease in recombination or enhanced separation of photoexcited electron‐hole pairs. This suggests the potential for more efficient charge carrier dynamics, which may be attributed to increased electron localization onto the 2HIC of the CRO‐Cg structure through an electron push‐pull effect. For CRO‐BtzTp‐Cg and CRO‐TtzTp‐Cg specifically, this phenomenon is expected to play a significant role in reducing the bandgap, which leads to a noticeable red‐shift in both the optical absorption spectrum and the PL emission.

The emission decay time was systematically examined using bi‐exponential decay kinetics to better understand the lifetime of photoinduced charge carriers in CRO‐BtzTp, CRO‐TtzTp, CRO‐BtzTp‐Cg, and CRO‐TtzTp‐Cg. The photoinduced charge carrier lifetime recorded for CRO‐BtzTp‐Cg (2.7 ns) and CRO‐TtzTp‐Cg (8.7 µs) were longer than that of CRO‐BtzTp (0.5 ns) and CRO‐TtzTp (8.4 µs). Differential carrier lifetimes between CRO‐TtzTp and CRO‐BtzTp, as well as CRO‐TtzTp‐Cg and CRO‐BtzTp‐Cg result from structural disparities. The *C*_3_+*C*_3_ symmetry in CRO‐TtzTp ensures a uniform electron distribution, fostering stable charge transport, while the *C*_2_+*C*_3_ symmetry in CRO‐BtzTp introduces potential asymmetry and heightened charge carrier recombination. Varied pore sizes play a role, with smaller pores in CRO‐TtzTp constraining encounters and recombination, and larger pores in CRO‐BtzTp enabling faster migration but increasing recombination probabilities. Moreover, Ttz's superior electron‐acceptor capability and the influence of end‐capping groups contribute to the extended carrier lifetime of TtzTp, attributed to its higher symmetry and coordinated interactions. Specifically, the proportion of short‐lived lifetime attributed to bulk recombination reduced, while the proportion of longer lifetime associated with surface recombination increased notably in CRO‐BtzTp‐Cg and CRO‐TtzTp‐Cg (Tables S2 and S3, Supporting Information). This trend suggests potential acceleration of bulk‐to‐surface electron transfer in CRO‐BtzTp‐Cg and CRO‐TtzTp‐Cg, therefore, a higher number of photoinduced carriers can be involved in the PEC water reaction process.

**Figure 3:**
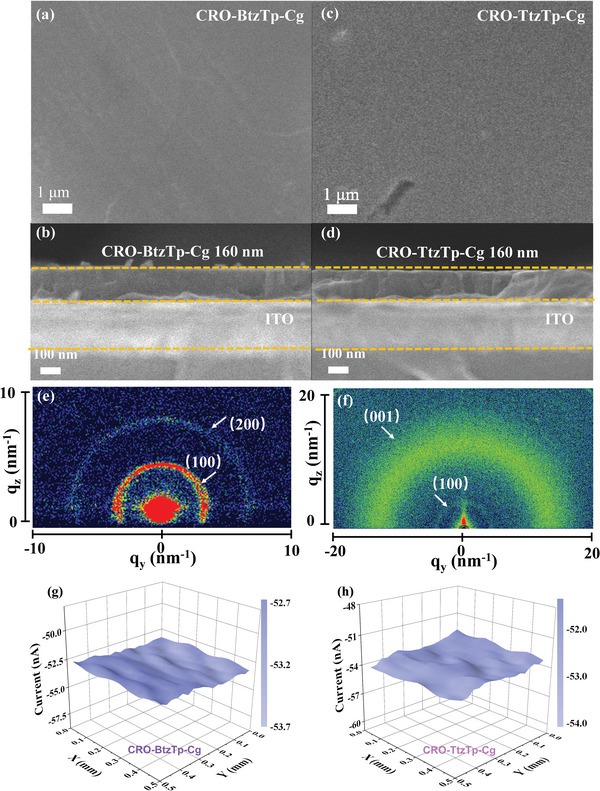
Morphology of the solution‐processed CRO‐BtzTp‐Cg and CRO‐TtzTp‐Cg nanomembranes. Top−down SEM morphology of spin‐coated a) CRO‐BtzTp‐Cg and d) CRO‐TtzTp‐Cg. Cross‐sectional SEM image of b) ITO/CRO‐BtzTp‐Cg and d) ITO/CRO‐TtzTp‐Cg after spin‐coating. 2D GIWAXS patterns of spin‐coated e) CRO‐BtzTp‐Cg and f) CRO‐TtzTp‐Cg nanomembranes on a SiO_2_/Si wafer. SECM images of g) CRO‐BtzTp‐Cg and h) CRO‐TtzTp‐Cg nanomembranes.

Scanning electron microscopy (SEM) was used to examine the film morphology produced via the spin‐coating method to validate the solution‐processed CRO‐BtzTp, CRO‐TtzTp, CRO‐BtzTp‐Cg, and CRO‐TtzTp‐Cg. It was observed that CRO‐BtzTp, CRO‐TtzTp, CRO‐BtzTp‐Cg, and CRO‐TtzTp‐Cg can produce more consecutive and uniform nanomembranes on ITO substrates (2.0 × 2.0 cm) (Figure S16, Supporting Information and **Figure** 3a,b) than Bulk‐BtzTp and Bulk‐TtzTp (Figure S17, Supporting Information). The SEM cross‐sectional images show that the CRO‐BtzTp‐Cg and CRO‐TtzTp‐Cg nanomembranes have a thickness of ≈160 nm and exhibit excellent adhesion to the substrate. We also have conducted Atomic Force Microscopy (AFM) measurements on the CRO‐BtzTp, CRO‐BtzTp‐Cg, CRO‐TtzTp, and CRO‐TtzTp‐Cg. Based on the AFM images in Figure S18, Supporting Information, the roughness of the spin‐coated CRO‐BtzTp, CRO‐BtzTp‐Cg, CRO‐TtzTp, and CRO‐TtzTp‐Cg films was determined to be 3.7, 3.7, 3.0, and 5.6 nm, respectively, indicating a relatively smooth and uniform surface. High‐quality nanomembranes prepared by solution‐processed CRO‐BtzTp, CRO‐TtzTp, CRO‐BtzTp‐Cg, and CRO‐TtzTp‐Cg are responsible for the efficient solar‐energy‐conversion devices.

Grazing‐incidence wide‐angle X‐ray scattering (GIWAXS) imaging was used to assess the crystallinity of CRO‐BtzTp‐Cg and CRO‐TtzTp‐Cg nanomembranes. Figure 3e shows the CRO‐BtzTp‐Cg nanomembrane reflection peaks with *q* values of 2.9 and 5.7 nm^‒1^, which are assigned to the (100) and (200) crystallographic planes, respectively. Similar results were seen with the CRO‐TtzTp‐Cg nanomembrane (Figure 3f), where two diffraction peaks appear at 4.9 and 14.9 nm^‒1^, which correspond to the (100) and (001) lattice planes. This indicates that CRO‐BtzTp‐Cg and CRO‐TtzTp‐Cg exhibit well‐defined crystalline structures.

We employed scanning electrochemical microscopy (SECM) in feedback mode to assess the flatness of the active layer surface. This involved adjusting the probe distance to approach the CRO layer without actual contact, followed by fixing the probe height. Then we performed planar scans in both the X and Y directions, covering an area of 0.5 × 0.5 mm, while simultaneously monitoring the current variations at different positions. As shown in Figure 3g,h, the current fluctuations for CRO‐BtzTp‐Cg and CRO‐TtzTp‐Cg remained within a narrow range of 3 nA, indicative of the steady‐state tip currents. It can be attributed to the absence of discernible cracks, holes, or any structural irregularities on the surface of the CRO‐BtzTp‐Cg and CRO‐TtzTp‐Cg films.

### 2.2 PEC‐HER Performance of Interlayer Heterojunction Devices

The PEC for Bulk‐BtzTp, CRO‐BtzTp, CRO‐BtzTp‐Cg, Bulk‐TtzTp, CRO‐TtzTp, and CRO‐TtzTp‐Cg photoelectrodes were evaluated using a three‐electrodes setup in an aqueous solution of Na_2_SO_4_ (0.1 mol L^‒1^, pH = 7) without a sacrificial agent. A three‐electrode configuration was used, i.e.: a reference‐electrode (Ag/AgCl, saturated KCl solution); a counter‐electrode using a platinum plate; a photocathode working electrode with a photoactive layer supported by an ITO substrate. The PEC‐HER activity was evaluated via linear sweep voltammetry (LSV), both in the absence of light and under simulated sunlight illumination through the substrate. The photocurrent of the photocathodes was negligible under illumination (Figure S19, Supporting Information) when fabricated by directly spinning coating Bulk‐BtzTp and Bulk‐TtzTp onto the ITO substrate. This may be attributed to the inability to process Bulk‐BtzTp and Bulk‐TtzTp, which can result in discontinuity of film or defects on the surface of the photoelectrode that lead to scattering and reflection of incident light, which reduces light absorption efficiency. The photocathodes prepared using CRO‐BtzTp, CRO‐BtzTp‐Cg, CRO‐TtzTp, and CRO‐TtzTp‐Cg on ITO glass indicated photocathodic current under light illumination. The onset potential for CRO‐BtzTp, CRO‐BtzTp‐Cg, CRO‐TtzTp, and CRO‐TtzTp‐Cg photocathodes was 0.97, 0.95, 0.96, and 1.11 V versus RHE, respectively. This confirms the possibility of producing solar‐driven electrons.

To develop strategies for inhibiting the recombination of photogenerated carriers and promoting the practical application of CROs in PEC, an investigation was conducted to assess the effect on the photocurrent response of incorporating an HTL and constructing an IHJ photoelectrode half‐device structure. Vapor‐deposited CuI was used as an HTL to enhance the performance of the PEC‐heterojunction device, due to its superior electron mobility, excellent optical transparency, high durability, and cost‐effectiveness.^[^ ^51^ ^]^ The results show that constructing a heterojunction with CuI and active materials (including Bulk‐BtzTp, CRO‐BtzTp, CRO‐BtzTp‐Cg, Bulk‐TtzTp, CRO‐TtzTp, and CRO‐TtzTp‐Cg) increases the photocurrent dramatically (Figure S20, Supporting Information). ITO/CuI/CRO‐BtzTp, ITO/CuI/CRO‐BtzTp‐Cg, ITO/CuI/CRO‐TtzTp and ITO/CuI/CRO‐TtzTp‐Cg show a Δ*J* between photocurrent and dark current of 4.7, 9.9, 14.8, and 25.6 µA cm^‒2^ at 0.4 V versus RHE, which is 1.6, 2.8, 4.5, and 5.4 times higher than that of ITO/CRO‐BtzTp, ITO/CRO‐BtzTp‐Cg, ITO/CRO‐TtzTp and ITO/CRO‐TtzTp‐Cg. The increase in photocurrent is attributed to the formation of a built‐in electric field in the IHJ photoelectrode half‐device, which effectively suppresses charge recombination in the interface of the heterojunction. It is suggested that half‐device fabrication is an effective approach to mitigating carrier recombination.

**Figure 4:**
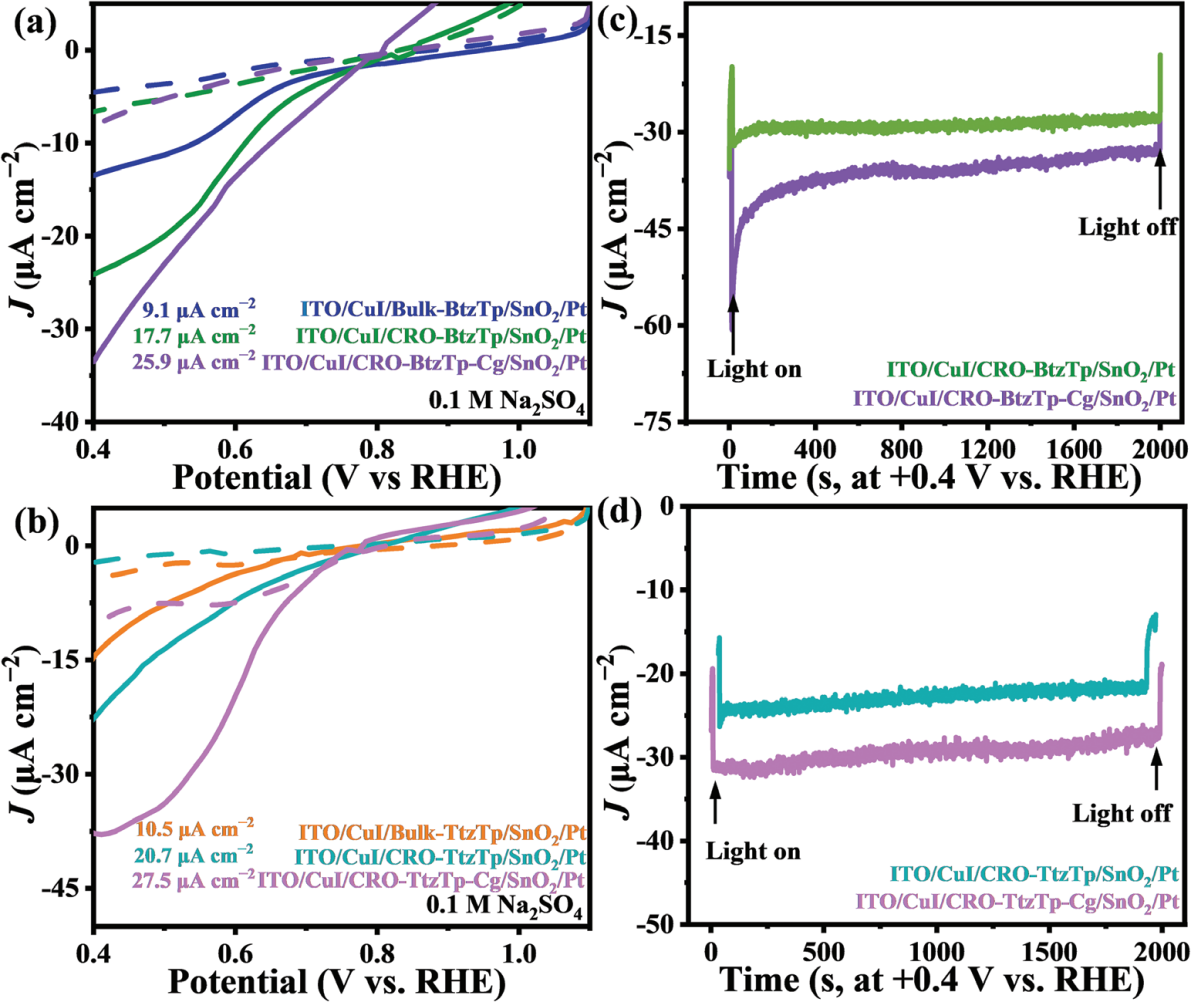
a,b) LSV curves of ITO/CuI/Bulk‐BtzTp/SnO_2_/Pt, ITO/CuI/CRO‐BtzTp/SnO_2_/Pt, ITO/CuI/CRO‐BtzTp‐Cg/SnO_2_/Pt, ITO/CuI/Bulk‐TtzTp/SnO_2_/Pt, ITO/CuI/CRO‐TtzTp/SnO_2_/Pt and ITO/CuI/CRO‐TtzTp‐Cg/SnO_2_/Pt. c,d) CA of ITO/CuI/CRO‐BtzTp/SnO_2_/Pt, ITO/CuI/CRO‐BtzTp‐Cg/SnO_2_/Pt, ITO/CuI/CRO‐TtzTp/SnO_2_/Pt and ITO/CuI/CRO‐TtzTp‐Cg/SnO_2_/Pt.

For purposes of studying electron transportation and the full‐device in PEC, SnO_2_ was selected as the ETL, as it can transport photogenerated electrons efficiently. This is due to its high electron mobility and low resistance, which provides a fast electron channel to promote the PEC.^[^ ^52^ ^]^ In addition, Pt nanoparticles were incorporated into the HER catalyst cover layer of the Bulk‐BtzTp, CRO‐BtzTp, CRO‐BtzTp‐Cg, Bulk‐TtzTp, CRO‐TtzTp, and CRO‐TtzTp‐Cg photocathodes, to provide catalytic active sites for HER. The optimized photocathode, comprising ITO/CuI/CRO‐TtzTp‐Cg/SnO_2_/Pt, exhibits a remarkable ∆*J* of 27.5 µA cm^‒2^ at 0.4 V versus RHE (**Figure** **4**a,b). This is 5.9 times higher than that achieved when using the ITO/CRO‐TtzTp‐Cg configuration. The ITO/CuI/CRO‐TtzTp‐Cg/Pt photoelectrode results show a slightly lower, but still considerable, photocurrent of 21.4 µA cm^‒2^ at 0.4 V versus RHE (Figure S21, Supporting Information), in comparison to the ITO/CuI/CRO‐TtzTp‐Cg/SnO_2_/Pt photoelectrode. This underscores the role of SnO_2_ as an effective electron‐collecting layer. The results obtained in this study provide experimental evidence for the capability of SnO_2_ to enhance the efficiency of electron collection in the photocathode structure. The chronoamperometry (CA) measurement shows that the four photocathodes included ITO/CuI/CRO‐BtzTp/SnO_2_/Pt, ITO/CuI/CRO‐BtzTp‐Cg/SnO_2_/Pt, ITO/CuI/CRO‐TtzTp/SnO_2_/Pt, and ITO/CuI/CRO‐TtzTp‐Cg/SnO_2_/Pt can be operated continuously at +0.4 V versus RHE for more than 50 mins (Figure 4c,d), which indicates good stability.

### 2.3 Investigation of the Mechanism

**Figure 5:**
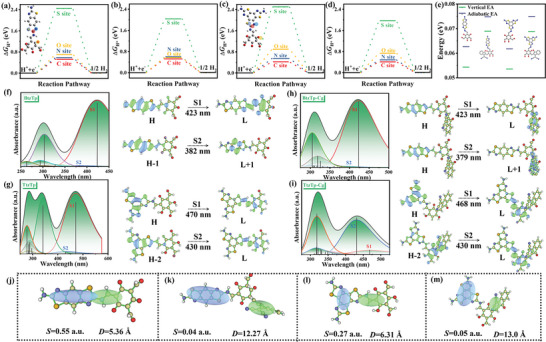
Gibbs free energy of a) CRO‐BtzTp; b) CRO‐BtzTp‐Cg; c) CRO‐TtzTp; d) CRO‐TtzTp‐Cg. d) Calculated electron affinity of CRO‐BtzTp, CRO‐BtzTp‐Cg, CRO‐TtzTp, and CRO‐TtzTp‐Cg. Calculated UV–vis absorption spectra and TD‐DFT calculated electronic transition of f) CRO‐BtzTp; g) CRO‐TtzTp; h) CRO‐BtzTp‐Cg; i) CRO‐TtzTp‐Cg. Excitation characteristics (S and D) of j) CRO‐BtzTp; k) CRO‐TtzTp; l) CRO‐BtzTp‐Cg; m) CRO‐TtzTp‐Cg.

The hydrogen evolution reaction (HER) pathway in CRO‐BtzTp, CRO‐BtzTp‐Cg, CRO‐TtzTp, and CRO‐TtzTp‐Cg was explored through Density Functional Theory (DFT) calculations to understand the intrinsic change caused by the capping group deeply. Gibbs free energy hydrogen adsorption (Δ*G*_H*_) on various sites of the CRO‐BtzTp, CRO‐BtzTp‐Cg, CRO‐TtzTp, and CRO‐TtzTp‐Cg were individually investigated based on previous research. As shown in **Figure** **5**a,c, a noticeable disparity in the Δ*G*_H*_ was seen following the hydrogenation of possible sites. The Δ*G*_H*_ observed at the sulfur sites indicates a high overpotential for HER. Conversely, the Δ*G*_H*_ values at the carbon sites in Btz and Ttz demonstrate minimal energy barriers, which suggests that incorporating Btz and Ttz units promotes optimal hydrogen adsorption. This is attributed to the uniform dispersion of sulfur and nitrogen heteroatoms within Btz and Ttz units, which induces charge redistribution and so enhances energetics for H_2_ formation. Importantly, there was a noticeable decrease in the energy barrier for the formation of adsorbed H* intermediates on potential sites in both CRO‐BtzTp‐Cg and CRO‐TtzTp‐Cg compared to that of CRO‐BtzTp and CRO‐TtzTp without capping groups (Figure 5b,d). These findings collectively indicate that the end‐capping strategy employed in CRO‐BtzTp‐Cg and CRO‐TtzTp‐Cg can effectively reduce the energy barrier to H_2_ formation. This is consistent with the results obtained in the PEC activity investigation.

Further investigation of the PEC mechanism of CRO‐BtzTp, CRO‐BtzTp‐Cg, CRO‐TtzTp, and CRO‐TtzTp‐Cg was done. The charge density calculated for the bands near the LUMO and Highest Occupied Molecular Orbital (HOMO) reveals the distribution of charges upon light irradiation‐induced excitation of CRO‐BtzTp, CRO‐BtzTp‐Cg, CRO‐TtzTp, and CRO‐TtzTp‐Cg. Figure S22, Supporting Information, shows that the LUMO and HUMO of CRO‐BtzTp are primarily contributed by the Tp and Btz moiety, while that of CRO‐TtzTp is mainly derived from the Tp and Ttz building blocks. Interestingly, the LUMO of CRO‐BtzTp‐Cg and CRO‐TtzTp‐Cg is primarily located in the capping group (2HIC), which indicates that the intrinsic change in LUMO energy level is caused by the end‐capping strategy. The Btz and Ttz moieties act as electron donors, providing electrons to counter the building block and inducing an electron‐deficient state.

The 2HIC units exhibited a relatively high electron density and functioned as electron acceptors. It appears that the exciton separation in the 2HIC units exhibits significantly prolonged survival and heightened stability. This outcome implies that effective charge separation can be achieved through charge stabilization at the electron acceptor, which enhances PEC activity. To assess the extent of stabilization of the excited electrons in CRO‐BtzTp, CRO‐BtzTp‐Cg, CRO‐TtzTp, and CRO‐TtzTp‐Cg, electron affinity (EA) was computed. The EA indicated the stability of the structure upon receiving additional electrons. Compared to CRO‐BtzTp and CRO‐TtzTp, the EA values of CRO‐BtzTp‐Cg and CRO‐TtzTp‐Cg are more positive, which can be attributed to the push‐pull effect induced by introducing an electron‐withdrawing capping group. It is suggested that the stability of the negative charge states of CRO‐BtzTp‐Cg and CRO‐TtzTp‐Cg exhibit an increasing trend relative to that of CRO‐BtzTp and CRO‐TtzTp. Enhancing PEC from CROs to CROs‐Cg is attributed to the charge stability achieved by using the end‐capping strategy, which effectively prolongs the lifetime of the charge.

We also investigated the charge transfer characteristics of CRO‐BtzTp, CRO‐TtzTp, CRO‐BtzTp‐Cg, and CRO‐TtzTp‐Cg employing time‐dependent Density Functional Theory (TD‐DFT) calculations.^[^ ^53^ ^]^ The calculated UV‐vis absorption spectra are shown in Figure 5f–i. The red‐shift observed in the calculated UV‐vis spectra of CRO‐BtzTp‐Cg and CRO‐TtzTp‐Cg following the introduction of a capping group is indicative of the effectiveness of the end‐capping strategy in broadening the absorbance range, which is consistent with measured UV‐vis results. The maximum absorption peak for CRO‐BtzTp and CRO‐BtzTp‐Cg originates from the electronic transitions of S_0_ → S_1_ and S_0_ → S_2_, respectively. The excited state S_1_ may be due to the dominant configuration of HOMO → LUMO, while the excited state S_2_ is primarily due to the dominant configuration of HOMO‒1 → LUMO+1. The contour surfaces of the frontier orbitals HOMO‒1, HOMO, LUMO, and LUMO+1 relevant to maximal absorption are shown in Figure 5f,h. Both the HOMO → LUMO and HOMO‒1 → LUMO+1 transitions show evidence of intramolecular charge transfer (ICT).

With CRO‐BtzTp, electron transfer occurs from the Btz segment to the Tp segment, whereas in CRO‐BtzTp‐Cg, electron transfer takes place from the Btz segment to the 2HIC segment. The UV‐vis absorption spectra and contour surfaces of the frontier orbitals for CRO‐TtzTp and CRO‐TtzTp‐Cg are relevant to maximal absorption and are shown in Figure 5g,i. The maximum absorption peak for CRO‐TtzTp and CRO‐TtzTp‐Cg arises from the electronic transitions of S_0_ → S_1_ and S_0_ → S_2_, respectively. The excited state S_1_ may be due to the dominant configuration of HOMO → LUMO, while the excited state S_2_ is primarily due to the dominant configuration of HOMO‒2 → LUMO. Similarly, ICT features are observed in both CRO‐TtzTp and CRO‐TtzTp‐Cg.

Multiwfn software was used to calculate the overlap integrals of hole‐electron distributions (S) and hole‐electron distribution derivatives (D)^[^ ^54^ ^]^ (Figure 5j–m). The smaller S/D values lead to a more prominent charge transfer. The calculated S/D values exhibit a decreasing trend in the order: CRO‐BtzTp (0.10), CRO‐TtzTp (0.04), CRO‐BtzTp‐Cg (0.003), and CRO‐TtzTp‐Cg (0.003). This indicates that charge transfer is more favorable in the presence of both CRO‐BtzTp‐Cg and CRO‐TtzTp‐Cg.

The results detailed above indicate that implementing the end‐capping strategy not only regulates the band structure but also establishes a built‐in D‒A heterojunction interface to facilitate exciton dissociation. CRO‐BtzTp and CRO‐TtzTp possess a distinctive molecular framework characterized by the photoelectrocatalytic activity units, solution processibility, π‐conjugated lattice, and highly aligned 1D channels. CRO‐BtzTp‐Cg and CRO‐TtzTp‐Cg deliver an extended spectral range by introducing the capping groups compared to CRO‐BtzTp and CRO‐TtzTp, which then facilitates the establishment of a seamlessly integrated system for coupling events involved in PEC. First, the conjugated frameworks play a pivotal role as a light‐harvesting system that facilitates the migration of excitons and promotes the transportation of the charge via the π clouds of the frameworks. This enhances efficient electron transfer in the required direction while minimizing the reverse recombination of the charges. Second, the open 1D channels and sub‐nanometer structures provide a multitude of interfaces to enhance mass transportation and create densely populated photoactive sites that significantly reduce the hopping distance for the charge carriers while promoting electron accumulation at reaction centers. Third, a continuous electron flow occurs at the interface as the electrons are transferred from light‐harvesting antennae (CRO‐BtzTp‐Cg and CRO‐TtzTp‐Cg) to active sites (Pt coating) via the ETL and HTL. The PEC devices of CRO‐BtzTp‐Cg and CRO‐TtzTp‐Cg incorporate built‐in mechanisms that form a light‐driven interlocking system, which facilitates seamless photochemical processes for PEC.

### 2.4 Improved PEC‐HER Performance of Bulk Heterojunction Devices

**Figure 6:**
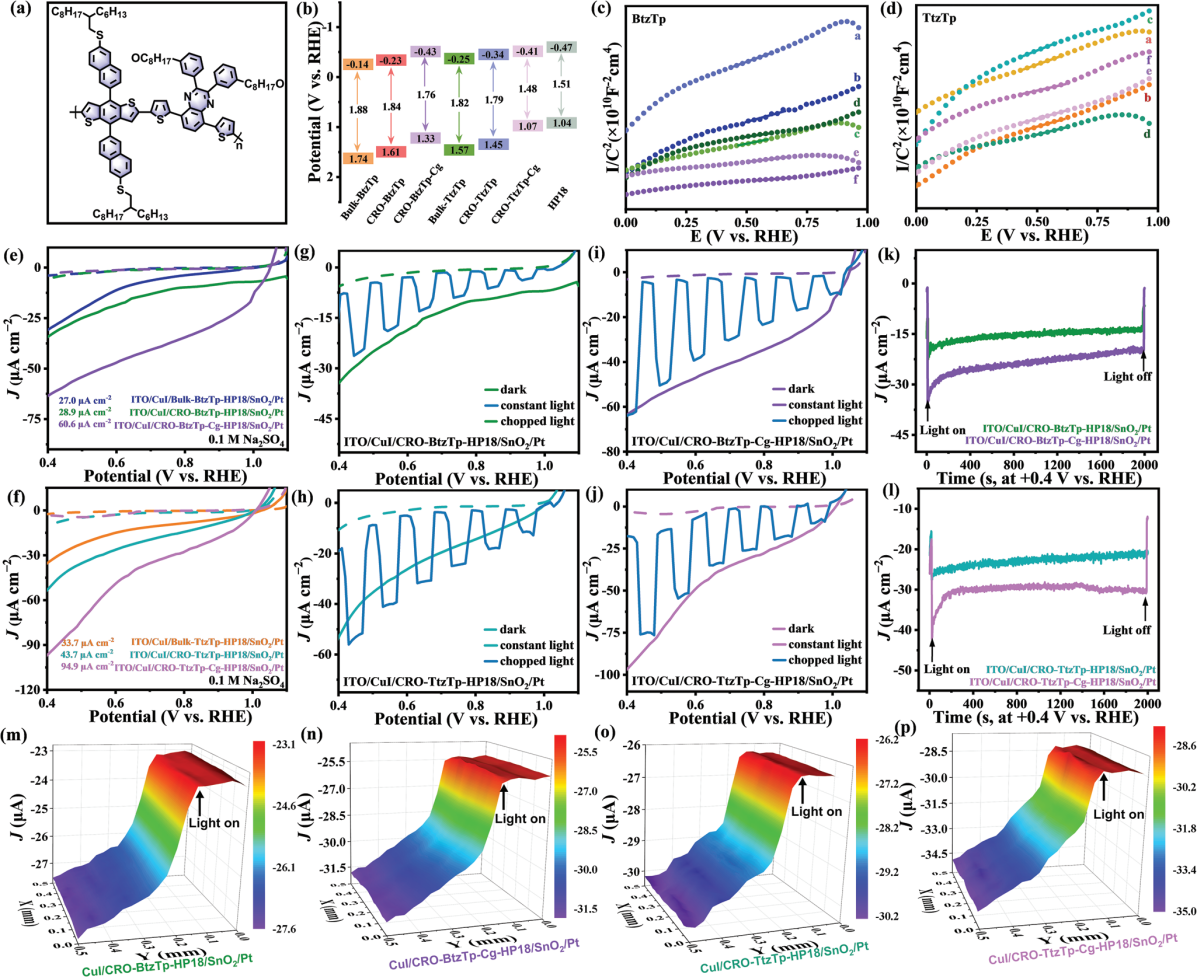
a) Chemical formula of the HP18. b) Energy levels of HP18, CROs and CROs‐CG. c) Mott–Schottky plots of a. Bulk‐BtzTp, b. Bulk‐BtzTp‐HP18, c. CRO‐BtzTp, d. CRO‐BtzTp‐HP18, e. CRO‐BtzTp‐Cg, f. CRO‐BtzTp‐HP18. d) Mott–Schottky plots of a. Bulk‐TtzTp, b. Bulk‐TtzTp‐HP18, c. CRO‐TtzTp, d. CRO‐TtzTp‐HP18, e. CRO‐TtzTp‐Cg, f. CRO‐TtzTp‐HP18. e,f) LSV curves of ITO/CuI/Bulk‐BtzTp‐HP18/SnO_2_/Pt, ITO/CuI/CRO‐BtzTp‐HP18/SnO_2_/Pt, ITO/CuI/CRO‐BtzTp‐Cg‐HP18/SnO_2_/Pt, ITO/CuI/Bulk‐TtzTp‐HP18/SnO_2_/Pt, ITO/CuI/CRO‐TtzTp‐HP18/SnO_2_/Pt, and ITO/CuI/CRO‐TtzTp‐Cg‐HP18/SnO_2_/Pt. g–j) LSV curves of ITO/CuI/CRO‐BtzTp‐HP18/SnO_2_/Pt, ITO/CuI/CRO‐BtzTp‐Cg‐HP18/SnO_2_/Pt, ITO/CuI/CRO‐TtzTp‐HP18/SnO_2_/Pt, and ITO/CuI/CRO‐TtzTp‐Cg‐HP18/SnO_2_/Pt under constant and chopped light. k,l) CA curves of ITO/CuI/CRO‐BtzTp‐HP18/SnO_2_/Pt, ITO/CuI/CRO‐BtzTp‐Cg‐HP18/SnO_2_/Pt, ITO/CuI/CRO‐TtzTp‐HP18/SnO_2_/Pt, and ITO/CuI/CRO‐TtzTp‐Cg‐HP18/SnO_2_/Pt. SECM images of m) ITO/CuI/CRO‐BtzTp‐HP18/SnO_2_/Pt, n) ITO/CuI/CRO‐BtzTp‐Cg‐HP18/SnO_2_/Pt, o) ITO/CuI/CRO‐TtzTp‐HP18/SnO_2_/Pt, and p) ITO/CuI/CRO‐TtzTp‐Cg‐HP18/SnO_2_/Pt under constant and chopped light.

The feasibility of engineering an interlayer heterojunction to enhance the performance of COF photoelectrodes was demonstrated by fabricating HTL/CROs/ETL/Pt IHJ devices using processable CROs and the spinning coating method. The results indicate the potential application of colloid COFs in PEC. It is conceivable that using BHJ structures to increase the area of the interfaces for charge separation in the photoactive layer may further enhance the photocurrent.^[^ ^55^ ^]^ Due to the smaller domain of the BHJ structures, carrier transport is facilitated via a shorter path and with reduced electron‐hole recombination, which results in faster electron transportation.^[^ ^56^ ^]^ Therefore, to further enhance the CROs and CRO‐Cg of the photocurrent, we developed a photoelectrode with a BHJ structure based on polymer donors (PDs) and CROs for PEC. The benzodithiophene‐based polymer is known as a high‐performance PD and was selected based on its HOMO energy level, which facilitates efficient electron excitation to the LUMO level.^[^ ^55^ ^]^ The chemical formula of the chosen PD (HP18) is shown in **Figure** **6**a. The energy levels of HP18, CROs, and CROs‐Cg are illustrated in Figure 6b. The Supporting Information provides the detailed synthesis step HP18.

In order to verify the role of the BHJ structures, Mott–Schottky analysis was done to determine the charge carrier density for PEC. The slope of the Mott‒Schottky plot is inversely proportional to the charge carrier density. Therefore, a smaller slope indicates a higher charge carrier density and faster charge transfer kinetics during the PEC process. CRO‐BtzTp‐Cg‐HP18 and CRO‐TtzTp‐Cg‐HP18 exhibit the poorest slope among the BtzTp‐ and TtzTp‐based photoelectrodes, as shown in Figures 6c and 5d. It is suggested that BHJ structures generate abundant charge carriers, which play a crucial role in enhancing the performance of PEC. The charge density differences of CRO‐BtzTp‐HP18, CRO‐TtzTp‐HP18, CRO‐BtzTp‐Cg‐HP18, and CRO‐TtzTp‐Cg‐HP18 were also simulated. As shown in Figure S25, Supporting Information, electronic accumulation takes place at the interface between HP18 and the active materials, which indicates facile charge interaction at the heterostructure of HP18 and the active materials.

It is evident from Figure S26, Supporting Information, that constructing a BHJ consisting of CROs and an HP18 donor polymer yields a notable positive shift in the onset overpotential relative to the CROs and HP18 (Figures S19 and S27, Supporting Information). This suggests that the CROs‐HP18 heterojunction enhances charge separation. Full details and discussions regarding the optimization of the device structure are provided in Figure S28, Supporting Information. In Figure 6e,f, ITO/CuI/CRO‐BtzTp‐HP18/SnO_2_/Pt, ITO/CuI/CRO‐BtzTp‐Cg‐HP18/SnO_2_/Pt, ITO/CuI/CRO‐TtzTp‐HP18/SnO_2_/Pt and ITO/CuI/CRO‐TtzTp‐Cg‐HP18/SnO_2_/Pt show a ∆*J* of 28.9, 60.6, 43.7, and 94.9 µA cm^‒2^ at 0.4 V versus RHE, which is 10.0, 16.8, 13.2, and 20.2 times higher than that of ITO/CRO‐BtzTp, ITO/CRO‐BtzTp‐Cg, ITO/CRO‐TtzTp and ITO/CRO‐TtzTp‐Cg. The incorporation of PD and active materials into a BHJ structure leads to a significant enhancement of the photocurrent.

In addition, we provided data on electrochemical active area (ECSA), incident‐photon‐to‐current efficiency (IPCE), and Faradaic efficiency for photoelectrodes to facilitate a fair comparison in ITO/CuI/CRO‐BtzTp‐HP18/SnO_2_/Pt, ITO/CuI/CRO‐BtzTp‐Cg‐HP18/SnO_2_/Pt, ITO/CuI/CRO‐TtzTp‐HP18/SnO_2_/Pt and ITO/CuI/CRO‐TtzTp‐Cg‐HP18/SnO_2_/Pt photoelectrodes. ECSA has a direct linear relationship with the double‐layer capacitance (*C*_dl_). *C*_dl_ was measured to evaluate the ECSA by CV in the non‐faradic region of 0.9–1.0 V versus RHE. The values of *C*_dl_ were calculated as 0.56, 0.70, 0.61, and 0.75 mF cm^‒2^ for ITO/CuI/CRO‐BtzTp‐HP18/SnO_2_/Pt, ITO/CuI/CRO‐BtzTp‐Cg‐HP18/SnO_2_/Pt, ITO/CuI/CRO‐TtzTp‐HP18/SnO_2_/Pt and ITO/CuI/CRO‐TtzTp‐Cg‐HP18/SnO_2_/Pt photoelectrodes (Figure S29a‒e, Supporting Information). The higher *C*_dl_ catalyst can provide a high reaction site density for adsorption, which is conducive to effective charge transfer.^[^ ^57^ ^]^

The IPCE serves as a crucial parameter for assessing the inherent PEC performance of photoelectrodes. Figure S30, Supporting Information displays the IPCE plots of CRO‐BtzTp, CRO‐BtzTp‐Cg, CRO‐TtzTp, and CRO‐TtzTp‐Cg. In contrast to the modest values of 0.37% for CRO‐BtzTp (at 550 nm), 0.46% for CRO‐TtzTp (at 550 nm), and 0.49% for CRO‐BtzTp‐Cg (at 550 nm), the peak IPCE of CRO‐TtzTp‐Cg stands out at an impressive 0.54% at 550 nm. This value represents a substantial improvement compared to the reported IPCE values at 550 nm for other COF photocathodes, such as BDT‐ETTA COFs (≈0.02%).^[^ ^25^ ^]^

The Faradic efficiency values mentioned are over 80% for different photoelectrodes (Figure S31, Supporting Information), including ITO/CuI/CRO‐BtzTp‐HP18/SnO_2_/Pt, ITO/CuI/CRO‐BtzTp‐Cg‐HP18/SnO_2_/Pt, ITO/CuI/CRO‐TtzTp‐HP18/SnO_2_/Pt, and ITO/CuI/CRO‐TtzTp‐Cg‐HP18/SnO_2_/Pt. This indicates that a significant proportion of the photogenerated electrons actively participate in the PEC water reduction process, leading to the generation of H_2_

Compared to the devices without HP18 (Figure S32, Supporting Information), the ITO/CuI/CRO‐BtzTp‐HP18/SnO_2_/Pt, ITO/CuI/CRO‐BtzTp‐Cg‐HP18/SnO_2_/Pt, ITO/CuI/CRO‐TtzTp‐HP18/SnO_2_/Pt, and ITO/CuI/CRO‐TtzTp‐Cg‐HP18/SnO_2_/Pt photoelectrodes show a more stable transient photocurrent, which can potentially lead to a more persistent and stable PEC. (See Figure 6g–j.) As shown in Figure 6k,i, the results of CA measurements demonstrate that the optimal photocathodes exhibit excellent stability, as they can be operated continuously at +0.4 versus RHE for more than 50 mins.

The localized PEC activity of the photoelectrodes was investigated by SECM in the dark and with light irradiation (Figure 6m–p). In situ monitoring of the photocatalytic activity of the ITO/CuI/CRO‐BtzTp‐HP18/SnO_2_/Pt, ITO/CuI/CRO‐BtzTp‐Cg‐HP18/SnO_2_/Pt, ITO/CuI/CRO‐TtzTp‐HP18/SnO_2_/Pt, and ITO/CuI/CRO‐TtzTp‐Cg‐HP18/SnO_2_/Pt photoelectrodes were conducted through generation–collection mode using redox couple. The ITO/CuI/CRO‐BtzTp‐HP18/SnO_2_/Pt, ITO/CuI/CRO‐BtzTp‐Cg‐HP18/SnO_2_/Pt, ITO/CuI/CRO‐TtzTp‐HP18/SnO_2_/Pt, and ITO/CuI/CRO‐TtzTp‐Cg‐HP18/SnO_2_/Pt show a dark current of 23.1, 25.3, 26.2, and 28.4 µA, respectively. The dark current of these photoelectrodes exhibits stable profiles, indicating the continuous nature of the photoelectrode surface. The photocurrent of ITO/CuI/CRO‐BtzTp‐HP18/SnO_2_/Pt, ITO/CuI/CRO‐BtzTp‐Cg‐HP18/SnO_2_/Pt, ITO/CuI/CRO‐TtzTp‐HP18/SnO_2_/Pt, and ITO/CuI/CRO‐TtzTp‐Cg‐HP18/SnO_2_/Pt is 27.6, 31.5, 30.2, and 35.0 µA, respectively. Under light irradiation, the surfaces of the ITO/CuI/CRO‐BtzTp‐HP18/SnO_2_/Pt, ITO/CuI/CRO‐BtzTp‐Cg‐HP18/SnO_2_/Pt, ITO/CuI/CRO‐TtzTp‐HP18/SnO_2_/Pt, and ITO/CuI/CRO‐TtzTp‐Cg‐HP18/SnO_2_/Pt photoelectrodes generate electron‐hole pairs, leading to an increase in the population of electrons available for the reduction of a redox mediator Fe^3+^. The enhancement in electron availability results in an improved tip photocurrent response. The heightened photocurrent indicates an increase in the generation of photogenerated charge carriers, resulting in an elevated electron concentration within the electron transport layer. As a result, more electrons can efficiently transfer to the catalytic layer, enhancing the efficiency of electron transfer. These photogenerated charge carriers move freely within the photoelectrodes when driven by an external circuit, providing additional electrons and holes for catalytic reactions.

The incorporation of HP18 into PEC devices results in an increased photocurrent and enhanced stability, which is attributed to the following factors: i) HP18 acts as an electron donor and provides additional electrons in the BHJ, which stabilizes the transient switching current. ii) Incorporation of the donor polymer enhances the performance of carrier transportation and mitigates non‐radiative recombination and loss in the BHJ. iii) The donor polymer enhances the separation and transfer of charge pairs generated upon light absorption, thereby promoting efficient charge pair migration to the catalyst surface for hydrogen production. iv) The CROs‐HP18 BHJ structures enable the expansion of the absorption spectrum, enhance light energy utilization efficiency, and facilitate efficient electron transportation and separation. The BHJ architecture offers a significantly increased interfacial area, facilitating effective interfacial connections between the CROs/CROs‐Cg and HP18. This results in improved transport efficiency of photogenerated carriers and the utilization rate of the catalysts, which ultimately enhances PEC hydrogen production.

## 3 Conclusion

In summary, we used molecular engineering to design solution‐processed benzothiazole‐based CRO colloids to fabricate panel‐type photoelectrodes in PEC‐HER. The obtained crystalline colloidal CROs and CROs‐Cg exhibit ink‐like printability, enabling the fabrication of large‐area panel‐type photoelectrode film with a smooth and homogeneous surface. The photoelectrodes prepared from CROs‐Cg colloids exhibit a more pronounced photocathodic current under light illumination in the absence of sacrificial agents. By employing heterojunction device engineering to design multicomponent photoelectrode structures that incorporate a BHJ (comprising a donating polymer and CRO‐Cg), an HTL, and an ETL, we effectively mitigated charge recombination in CRO‐Cg, which resulted in significant enhancement of photocurrent density. The optimized ITO/CuI/CRO‐BtzTp‐Cg‐HP18/SnO_2_/Pt and ITO/CuI/CRO‐TtzTp‐Cg‐HP18/SnO_2_/Pt photoelectrodes showed a photocurrent of 60.6 and 94.9 µA cm^‒2^ at 0.4 V versus RHE, which is 40.4 and 47.5 times higher than that of ITO/Bulk‐BtzTp and ITO/Bulk‐TtzTp. Significantly, this work addresses the bottleneck issue arising from the limited processability of COFs in optoelectronic devices. The resulting high‐quality nanomembranes and multicomponent photoelectrode designs help to improve our understanding of the intricacies of photoelectrode preparation and functional interplays among various components. This breakthrough sets the stage for COF applications in next‐generation semiconductor devices.

## Conflict of Interest

The authors declare no conflict of interest.

## Supporting information

Supporting Information

## Data Availability

Research data are not shared.
