# Supplementary material for: Molecular and Heterojunction Device Engineering of Solution‐Processed Conjugated Reticular Oligomers: Enhanced Photoelectrochemical Hydrogen Evolution through High‐Effective Exciton Separation
Source: Adv Sci (Weinh). 2024 Mar 7;11(18):2308535. doi: 10.1002/advs.202308535 (PMC11095168; doi:10.1002/advs.202308535)
Supplement: Supplementary file 1 — Supporting Information [file ADVS-11-2308535-s001.pdf]

ADVANCED SCIENCE
Open Access

Supporting Information

for *Adv. Sci.*, DOI 10.1002/advs.202308535

Molecular and Heterojunction Device Engineering of Solution-Processed Conjugated Reticular Oligomers: Enhanced Photoelectrochemical Hydrogen Evolution through High-Effective Exciton Separation

Boying Zhang, Huimin Gao, Yazhou Kang, Xiaoming Li, Qing Li, Pengda Zhai, Diane Hildebrandt, Xinying Liu*, Yue Wang and Shanlin Qiao**

Supporting information

Molecular and Heterojunction Device Engineering of Solution-Processed Conjugated Reticular Oligomers: Enhanced Photoelectrochemical Hydrogen Evolution through High-Effective Exciton Separation

Boying Zhang‡, Huimin Gao‡, Yazhou Kang, Xiaoming Li, Qing Li, Pengda Zhai, Diane Hildebrandt, Xinying Liu*, Yue Wang, Shanlin Qiao**

B. Zhang, H. Gao, Y. Kang, X. Li, Dr. Q. Li, P. Zhai, Dr. Y. Wang, Prof. S. Qiao
College of Chemistry and Pharmaceutical Engineering, Hebei University of Science and Technology, Shijiazhuang, 050018, China
E-mail: ccpeslqiao@hebust.edu.cn (S. Qiao)
iccaslq@hebust.edu.cn (Q. Li)
B. Zhang
Department of Chemical Engineering, Faculty of Engineering and the Built Environment, University of Johannesburg, Doornfontein, 2028, South Africa

Prof. D. Hildebrandt
Department of Chemical and Biochemical Engineering, Rutgers University, Piscataway, New Jersey, 08854, United States

Prof. X. Liu
Institute for Catalysis and Energy Solutions, University of South Africa, Florida, 1709, South Africa
E-mail: liux@unisa.ac.za (X. Liu)

‡ These authors contributed equally to this work

Table of Content

Section 1. Materials and methods

1.1 Materials

1.2 Material characterizations

1.3 Photoelectrodes fabrication

1.4 Photoelectrochemical measurements

Section 2. Synthetic Procedures

2.1 Synthesis of CRO-BtzTp

2.2 Synthesis of CRO-TtzTp

2.3 Synthesis of Bulk-BtzTp

2.4 Synthesis of Bulk-TtzTp

2.5 Synthesis of CRO-BtzTp-Cg

2.6 Synthesis of CRO-TtzTp-Cg

2.7 Synthesis of Pt co-catalyst

2.8 Synthesis HP18

Section 3. Supplementary figures

Section 4. Theoretical calculations

Section 1. Materials and methods

1.1 Materials

The purchased chemical materials listed below were used without any additional purification. Triformylphloroglucinol (Tp) and *bis*-benzothiazole diamine (Btz) and *tris*-benzothiazole triamine (Ttz) were purchased from Jilin Chinese Academy of Sciences–Yanshen Technology Co. Ltd. *N*, *N*-dimethylformamide, ethanol, acetone, chloroform, dimethyl sulfoxide (DMSO), hydrogen chloride solution (HCl) and pyridine were purchased from Damao chemical reagent factory (Tianjin, China). Mesitylene, dioxane, *N*-methylpyrrolidine (NMP), acetic acid, sodium dodecyl sulfate (SDS), hexadecyltrimethylammonium bromide (CTAB), dicyanomethyleneindianone (2HIC), polyvinylpyrrolidone (PVP, K15) and $H_2PtCl_6{\cdot}6H_2O$ were obtained from Aladdin Industrial Corporation (Shanghai, China).

1.2 Material characterizations

The equipment used to do the characterization work is as listed below: X-ray diffraction (XRD) was performed on a Rigaku D/MAX-2500 diffractometer with Cu Kα radiation (λ= 1.5406 Å). Solid ultraviolet (UV) spectra were recorded using a UV–vis Spectrometer Lambda 750S (Perkin Elmer, Inc., USA) at room temperature. Fourier transform infrared (FT–IR) spectra were recorded using a Thermo Scientific Nicolet iS10 spectrometer. A scanning electron microscope (SEM) (JEOL) was used to record the morphology of the samples. Transmission electron microscope (TEM) and high-resolution TEM images were recorded on a transmission electron microscope (JEOL, JEM–2100). X-ray photoelectron spectroscopy (XPS) data were collected using a Thermo Scientific K-Alpha spectrometer. Photoluminescence spectroscopy was recorded using a F97XP fluorescence spectrophotometer obtained from Shanghai Lingguang Technology Co. Ltd. The contact angle data was collected using a contact angle meter obtained from Data physics OCA20. Electron paramagnetic resonance (EPR) spectra was recorded using a Bruker EMX plus spectrometer. A scanning electrochemical microscopy (SECM) was used to measure the localized PEC activity of the photoelectrodes were investigated

1.3 Photoelectrodes fabrication

The ITO-coated glass substrates underwent a sequential sonication process with deionized water (20 minutes, 5 times), followed by acetone (20 minutes) and isopropanol (20 minutes), before being dried using compressed air.

Firstly, a 5 nm layer of CuI was deposited onto ITO using the evaporation method. Secondly, the CRO-BtzTp, CRO-BtzTp-Cg, CRO-TtzTp, and CRO-TtzTp-Cg layers were prepared by spin-coating a 3 mg mL^{-1} colloidal solution in chloroform with 30 μL of Nafion 117 at 1500 rpm for 60 seconds each, followed by a thermal annealing at 80 °C for 15 min in the air. For the bulk heterojunctions, the active material was mixed with the donor polymer in chloroform at a concentration of 3 mg mL^{-1}. Thirdly, the 5 nm layer of SnO_2 was deposited onto CRO-BtzTp and so on. Pt catalyst overlayer was prepared by adding 30 μL Nafion 117 solution in 1 mL Pt nanoparticle dispersion as binder, and then spin-coated the solution onto CRO-BtzTp, CRO-BtzTp-Cg, CRO-TtzTp, and CRO-TtzTp-Cg films or SnO_2 at 1500 rpm for 60 s. After the coating, the films were annealed at 80 °C for 15 min to remove the solvent residual.

For comparative purposes, photoelectrodes of BtzTp and TtzTp were also prepared using the aforementioned method.

1.4 Photoelectrochemical measurements

Photoelectrochemical measurements were carried out in a conventional three-electrode configuration powered by an electrochemical workstation (Princeton, U.S.). A standard

three-electrode cell consist of a working electrode, a platinum sheet as the counter electrode, and an Ag/AgCl (Sat. KCl) reference electrode. The photocathode was used as the working electrode with an active area of 0.25 cm^2. Simulated 1 sun illumination (AM 1.5G, 100 mW cm^{-2}) was provided by a Xe light (Perfect light). PEC-HER performance evaluation was performed by illuminating from the substrate side. Electrolyte (0.1 M) was prepared by dissolving Na_2SO_4 in ultrapure water. The electrolyte pH was determined by a pH meter, calibrated with standard pH buffers. The electrolyte was purged with N_2 for 15 min prior to the measurements. The applied potential vs. Ag/AgCl was converted to RHE potentials by the following equation:

$$E_{\mathrm{RHE}} = E_{\mathrm{Ag/AgCl}} + 0.197\ \mathrm{V} + 0.0591 * \mathrm{pH} \qquad \text{Equation 1}$$

The electrochemical measurements were performed on a CHI760e electrochemical workstation (Chenhua Instrument, Shanghai, China) using a standard three-electrode cell with a working electrode, a platinum sheet as the counter electrode, and an Ag/AgCl reference electrode. The working electrodes were prepared by the following steps: The sample, weighing 4 mg, was dispersed in a mixture of 750 μL ultrapure water and 250 μL ethanol. Then, 30 μL Nafion 117 (5% in a mixture of lower aliphatic alcohols and water) was added to the solution followed by ultrasonic treatment for 30 min to form a homogeneous solution. Then, the above solution was dropped onto the precleaned 1 × 1 cm ITO glass electrode, followed by air-drying before measurement. Electrochemical impedance (EIS) was measured in aqueous electrolyte solution (0.5 M Na_2SO_4) in the frequency range from 0.1 Hz to 100 K at the open-circle potential.

The transient photocurrent responses were measured in 0.5 M Na_2SO_4 electrolyte aqueous solution under visible-light illumination.

Mott–Schottky (M–S) plots were measured in 0.2 M Na_2SO_4 electrolyte aqueous solution.

Section 2. Synthetic Procedures

2.1 Synthesis of CRO-BtzTp

Tp (0.048 mmol, 10 mg) was dissolved in dimethyl sulfoxide (DMSO, 0.4 mL). This solution was added dropwise to an aqueous solution of cetyltrimethylammonium ammonium bromide (CTAB, 0.1 M, 30 mL) under ultrasonication. An aqueous solution of sodium dodecyl sulfate (SDS, 0.1 M, 0.93 mL) was then added. Btz (0.071 mmol, 16mg) was dissolved in HCl aqueous solution (0.1M, 1.5 mL). This solution was added dropwise to an aqueous solution of CTAB (0.1 M, 30 mL) under ultrasonication. An aqueous solution of SDS (0.1 M, 0.93 mL) was then added. The two resulting aqueous solutions were then mixed. The mixed solution was allowed to react at 30 °C for 3 days.

2.2 Synthesis of CRO-TtzTp

Tp (0.024 mmol, 5 mg) was dissolved in 0.8 mL of dimethyl sulfoxide (DMSO). This solution was added dropwise to an aqueous solution of cetyltrimethylammonium ammonium bromide (CTAB, 0.1 M, 50 mL) under ultrasonication. An aqueous solution of sodium dodecyl sulfate (SDS, 0.1 M, 1.54 mL) was then added. Ttz (0.024 mmol, 7mg) was dissolved in 0.8 mL of DMSO. This solution was added dropwise to an aqueous solution of CTAB (0.1 M, 50 mL) under ultrasonication. An aqueous solution of SDS (0.1 M, 1.54 mL) was then added. The two resulting aqueous solutions were then mixed, and 5 mL of acetic acid was added to the mixture. The mixed solution was allowed to react at 30 °C for 3 days.

2.3 Synthesis of Bulk-BtzTp

Tp (0.067 mmol,14 mg) and Btz (0.1 mmol,22mg) were weighted into a Pyrex tube (volume of ca. 10 mL). The mixture was dissolved in 2 mL of mesitylene/dioxane (1:1 v/v) and sonicated for 10 mins. After the aqueous acetic acid (8 M, 0.4 mL) was added, the solution was sonicated for 5 mins to ensure uniform dispersion. The Pyrex tube was degassed by means of three freeze-pump-thaw cycles and then flame-sealed. The tube was placed in an oven at 120 °C for 3 days. When the reaction time was up, the ampoule was cooled to room temperature and opened. The product was collected centrifugally and cleaned with DMF and acetone. The powder was dried in an oven at

100 °C under vacuum overnight.

2.4 Synthesis of Bulk-TtzTp

Tp (0.033 mmol, 7 mg) and Ttz (0.033 mmol, 9.8 mg) were weighted into a Pyrex tube (volume of ca. 10 mL). The mixture was dissolved in 1 mL of NMP/mesitylene (1:3 v/v) and sonicated for 10 mins. After the aqueous acetic acid (6 M, 0.1 mL) was added, the solution was sonicated for 5 mins to ensure uniform dispersion. The Pyrex tube was degassed by means of three freeze-pump-thaw cycles and then flame-sealed. The tube was placed in an oven at 120 °C for 3 days. When the reaction time was up, the ampoule was cooled to room temperature and opened. The product was collected centrifugally and cleaned with DMF and acetone. The powder was dried in an oven at 100 °C under vacuum overnight.

2.5 Synthesis of CRO-BtzTp-Cg

CRO-BtzTp (4 mg) and 2HIC (9.32 mg) were weighted into a Pyrex tube. The mixture was dissolved in a solution of chloroform (4 mL) and pyridine (0.83 mL). The Pyrex tube was reacted in a microwave reactor at 200 W and 150 ºC for 20 min.

2.6 Synthesis of CRO-TtzTp-Cg

CRO-TtzTp (4 mg) and 2HIC (9.32 mg) were weighted into a Pyrex tube. The mixture was dissolved in a solution of chloroform (4 mL) and pyridine (0.83 mL). The Pyrex tube was reacted in a microwave reactor at 200 W and 150 ºC for 20 min.

2.7 Synthesis of Pt co-catalyst

PVP (1.31×10^{-3} mol, 0.146 g) and $H_2PtCl_6\cdot6H_2O$ (6.55×10^{-5} mol, 33 mg) were dissolved in a mixture of water (50.4 mL) and ethanol (5.6 mL), followed by refluxing for three hours. After cooling to room temperature, the resulting solution was subjected to rotary evaporation to yield 5 mL of black liquid.

2.8 Synthesis of HP18

The monomer 5,8-dibromo-2,3-bis(3-(2-ethylhexyloxy)phenyl)-quinoxaline (M2) was purchased from Solarmer Materials Inc. The monomer M1 were synthesized following procedures reported literature.[1] All other chemical reagents were used as received.

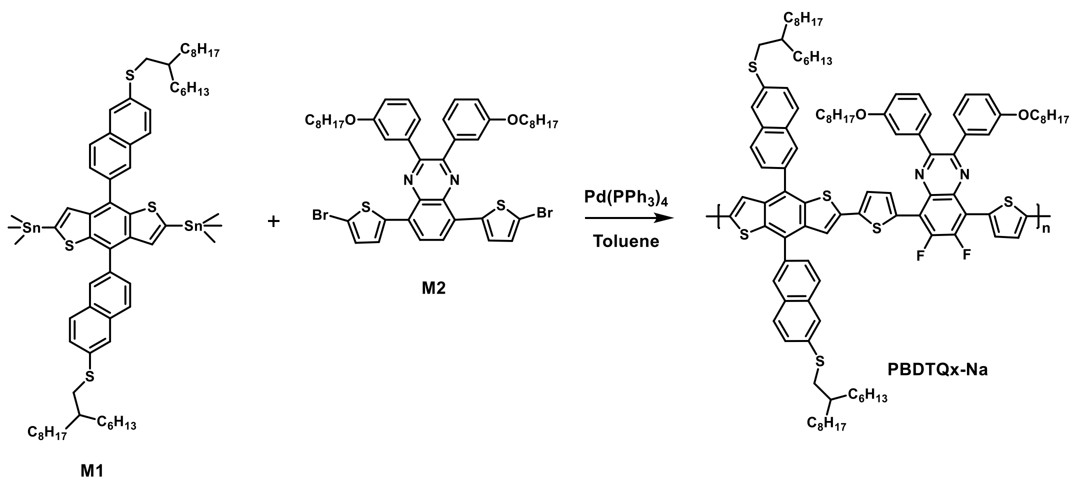

M1 (128.1 mg, 0.1 mmol) and M2 (86.1 mg, 0.1 mmol) were dissolved in degassed toluene (5 mL), $Pd(PPh_3)_4$ (5 mg) was added and purged with argon for 10 min, and then the mixture was vigorously stirred at 110 °C for 24 h under argon. After cooling down to room temperature, the solution was poured into methanol. The polymer was collected by filtration and Soxhlet-extracted in order with methanol, hexane and chloroform, the chloroform solution was concentrated to a small volume and precipitated by pouring into methanol again. Finally, the polymer was collected by filtration and dried under vacuum at 50 °C overnight and afforded PBDTQx-Na in 80% yield.

Section 3. Supplementary figures

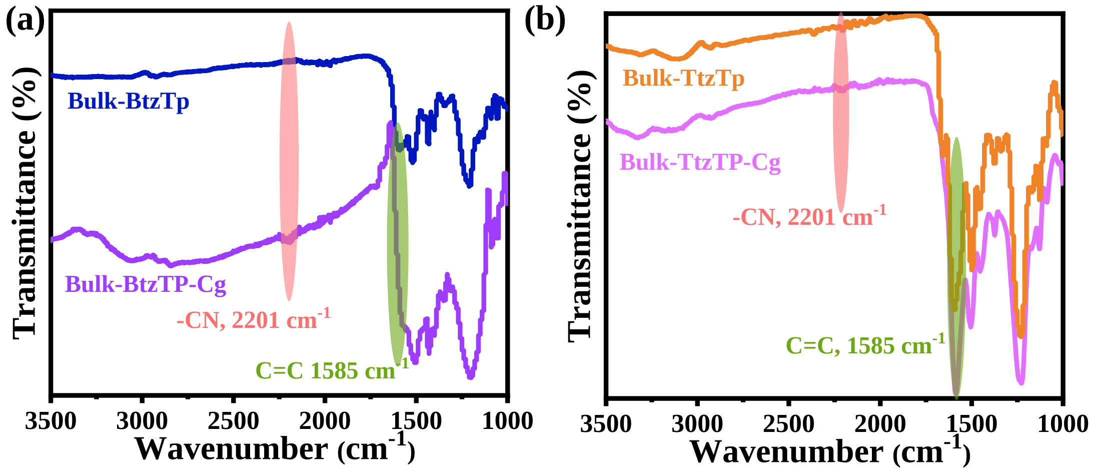

Figure S1. FT–IR spectra of (a) Bulk-BtzTp-Cg and (b) Bulk-TtzTp-Cg.

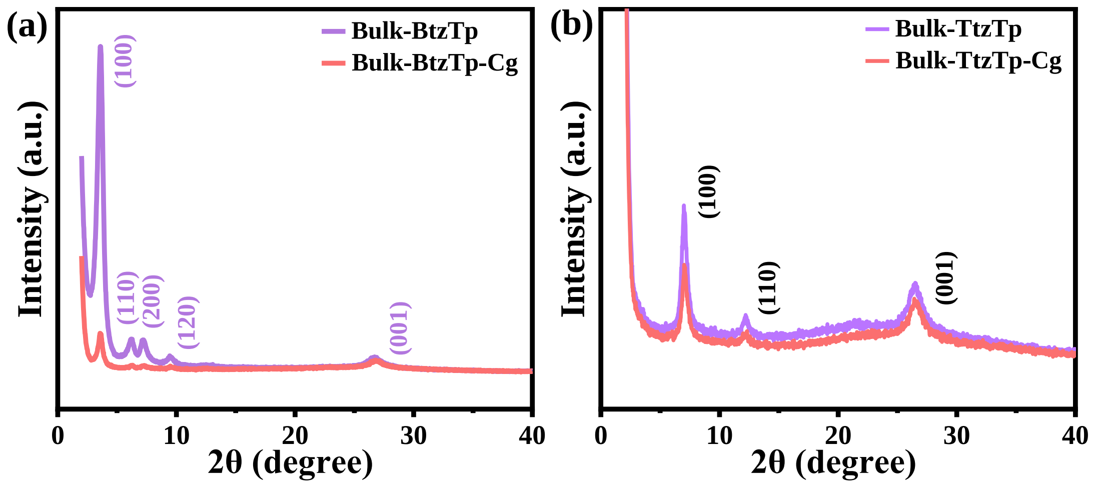

Figure S2. P-XRD patterns of (a) Bulk-BtzTp-Cg and (b) Bulk-TtzTp-Cg.

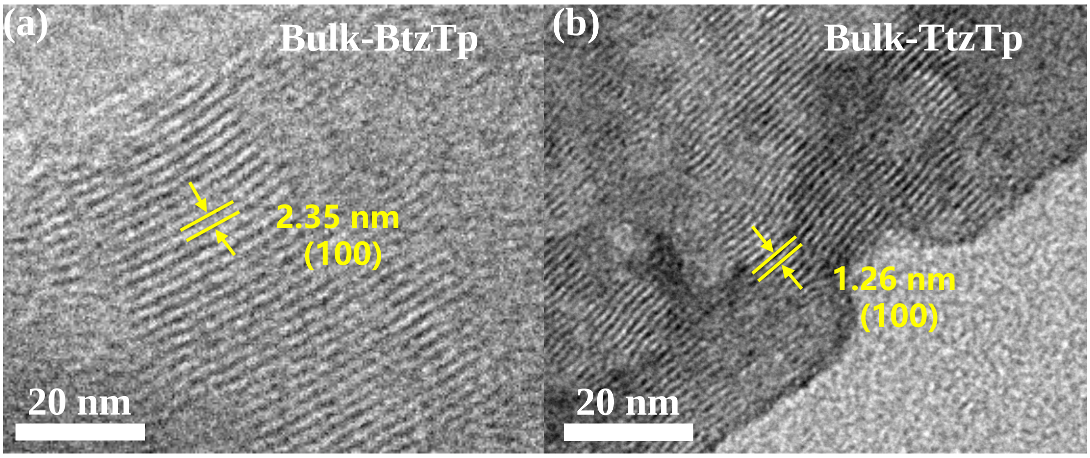

Figure S3. HR-TEM images of Bulk-BtzTp and Bulk-TtzTp.

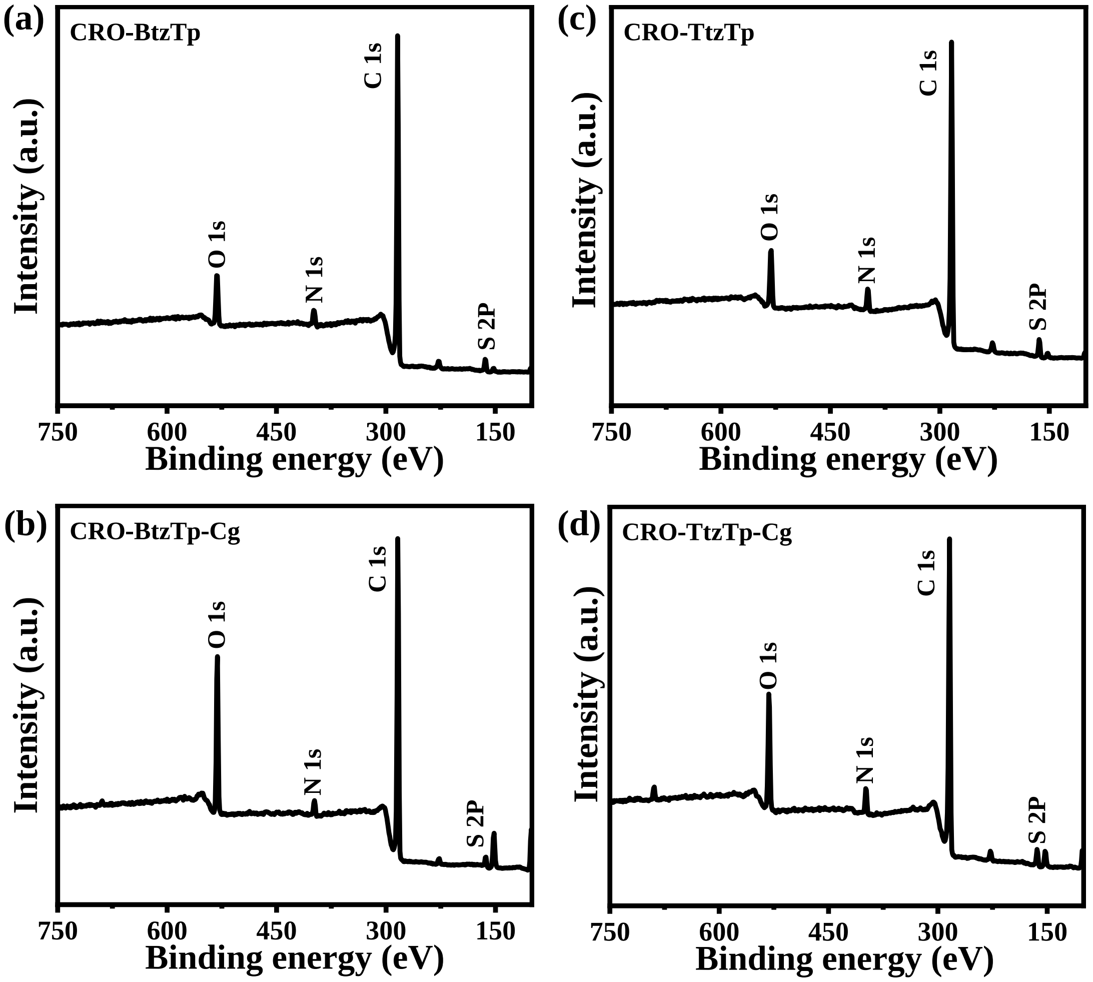

Figure S4. XPS spectra of (a) CRO-BtzTp, (b) CRO-BtzTp-Cg, (c) CRO-BtzTp, and (d) CRO-BtzTp-Cg.

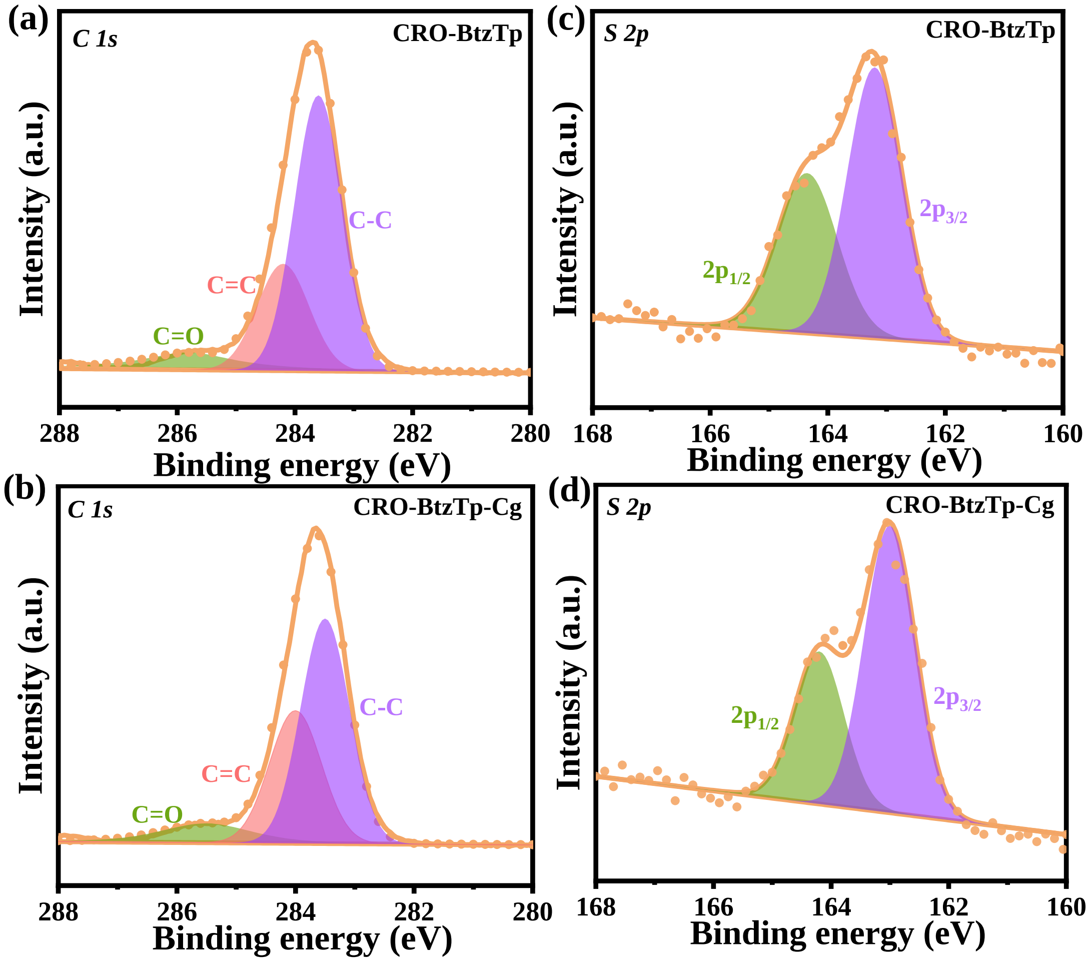

Figure S5. Deconvoluted C 1*s* of (a) CRO-BtzTp and (b) CRO-BtzTp-Cg. Deconvoluted S 2*p* of (g) CRO-BtzTp and CRO-BtzTp-Cg.

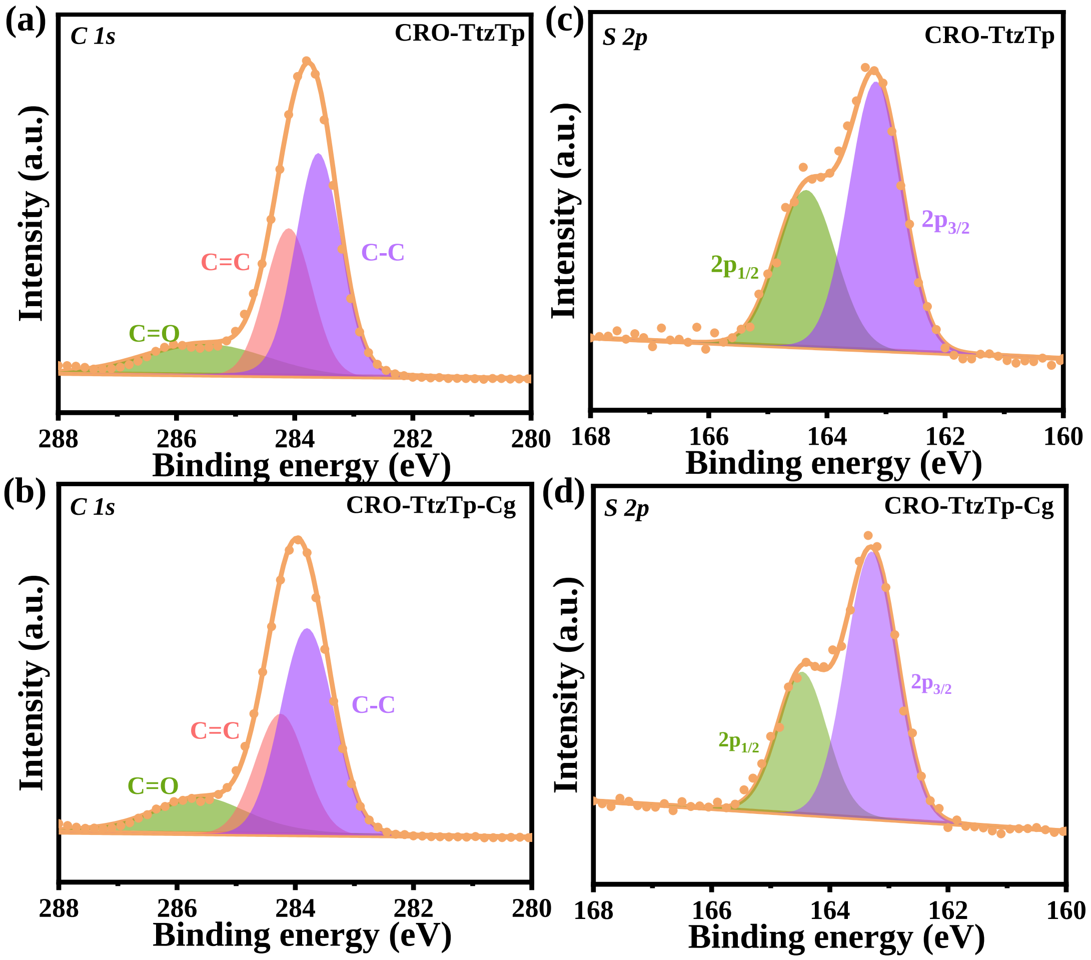

Figure S6. Deconvoluted C 1*s* of (a) CRO-TtzTp and (b) CRO-TtzTp-Cg. Deconvoluted S 2*p* of (g) CRO-TtzTp and CRO-TtzTp-Cg.

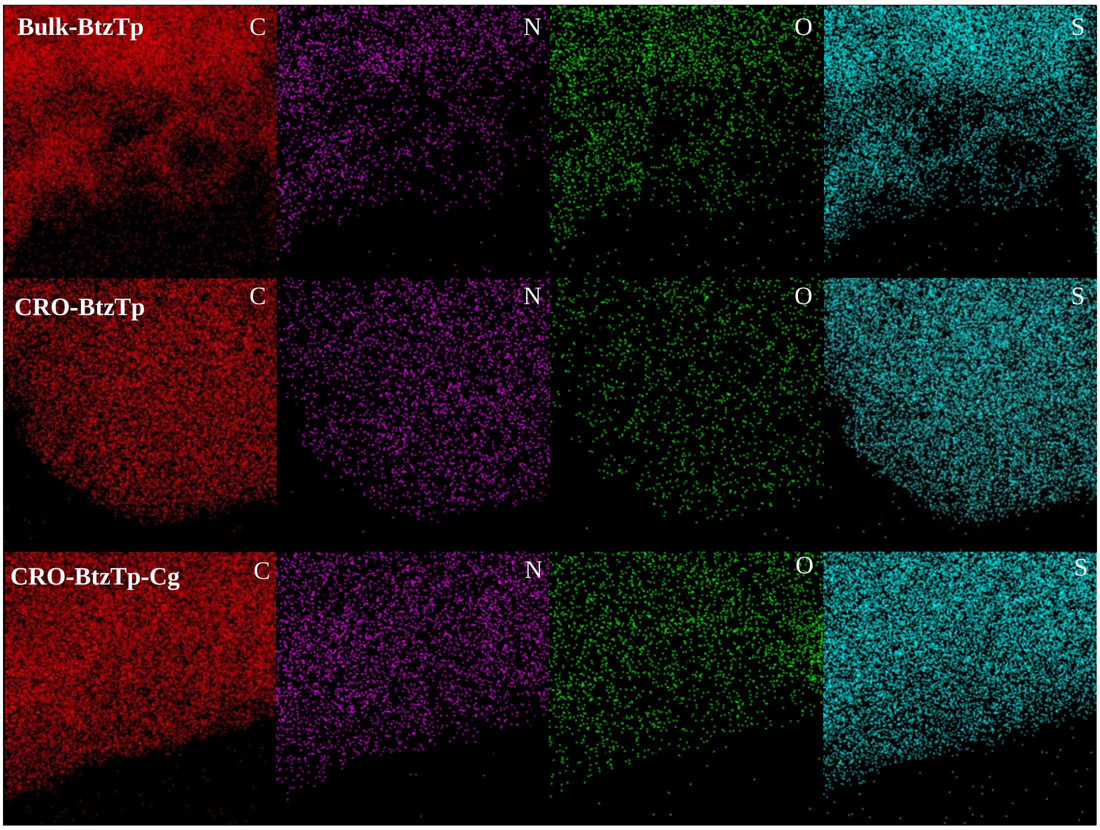

Figure S7. TEM-EDS elemental mapping images of Bulk-BtzTp, CRO-BtzTp, and CRO-BtzTp-Cg.

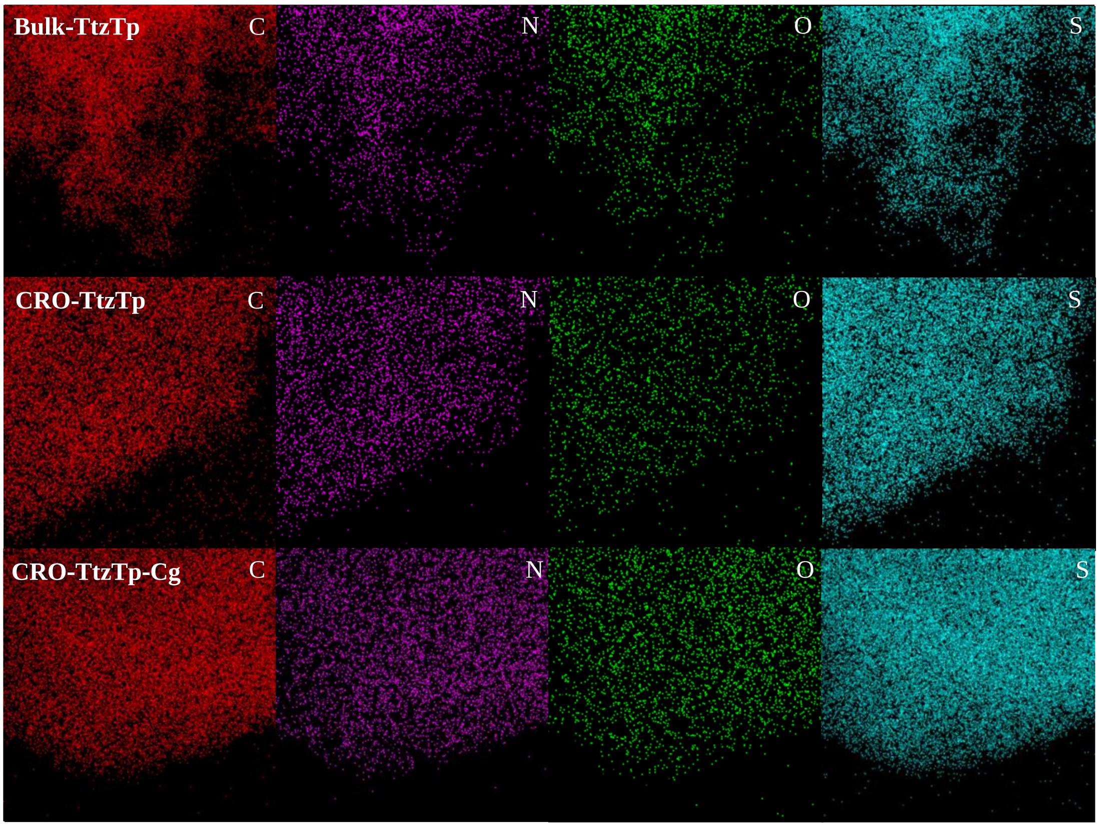

Figure S8. TEM-EDS elemental mapping images of Bulk-TtzTp, CRO-TtzTp, and CRO-TtzTp-Cg.

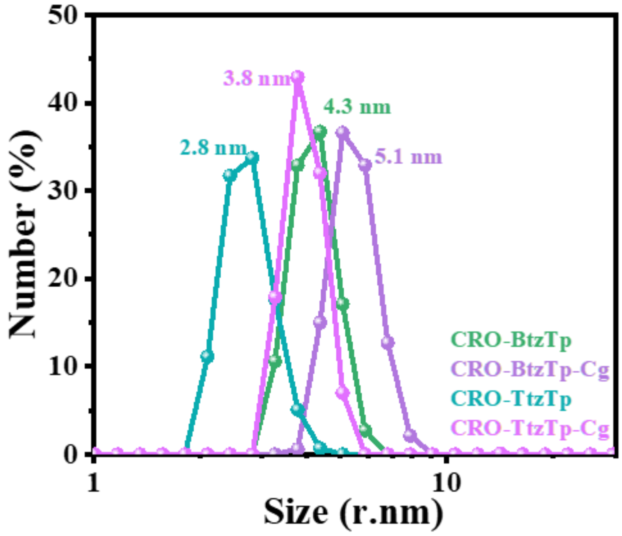

Figure S9. DLS analysis of CRO-BtzTp, CRO-BtzTp-Cg, CRO-TtzTp, and CRO-TtzTp-Cg.

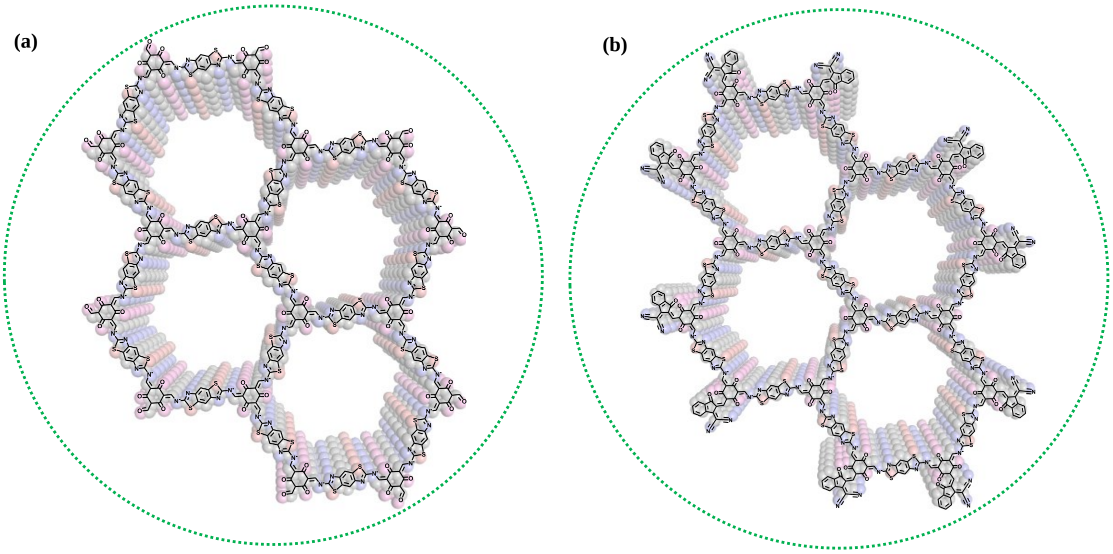

Figure S10. Notional molecular structure of (a) CRO-BtzTp and (b) CRO-BtzTp-Cg.

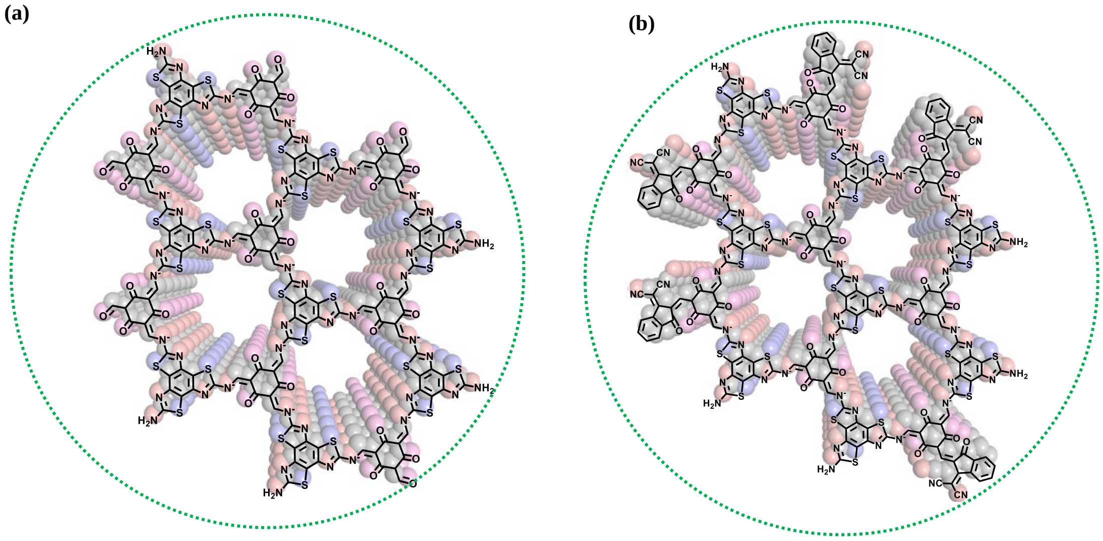

Figure S11. Notional molecular structure of (a) CRO-TtzTp and (b) CRO-TtzTp-Cg.

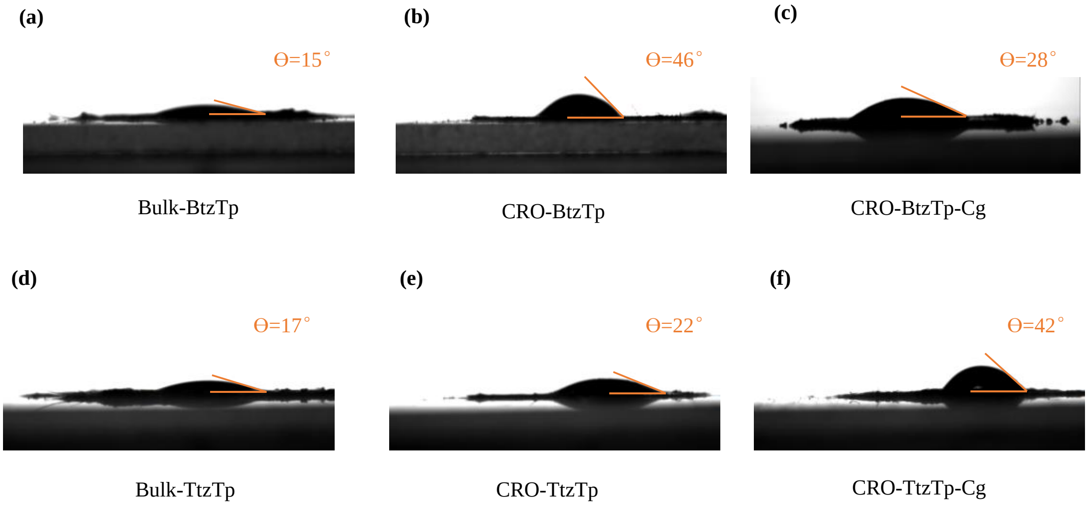

Figure S12. Contact angle of Bulk-BtzTp, CRO-BtzTp, CRO-BtzTp-Cg, Bulk-TtzTp, CRO-TtzTp, and CRO-TtzTp-Cg.

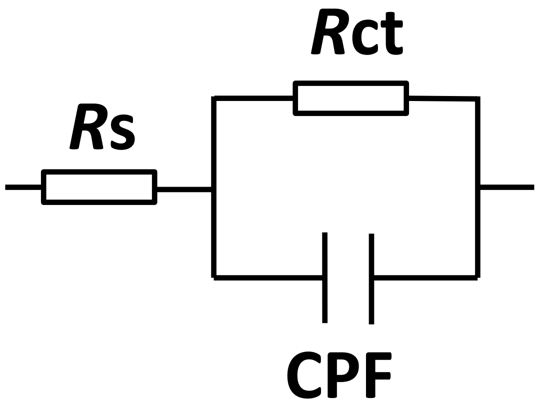

Figure S13. Equivalent circuit diagram.

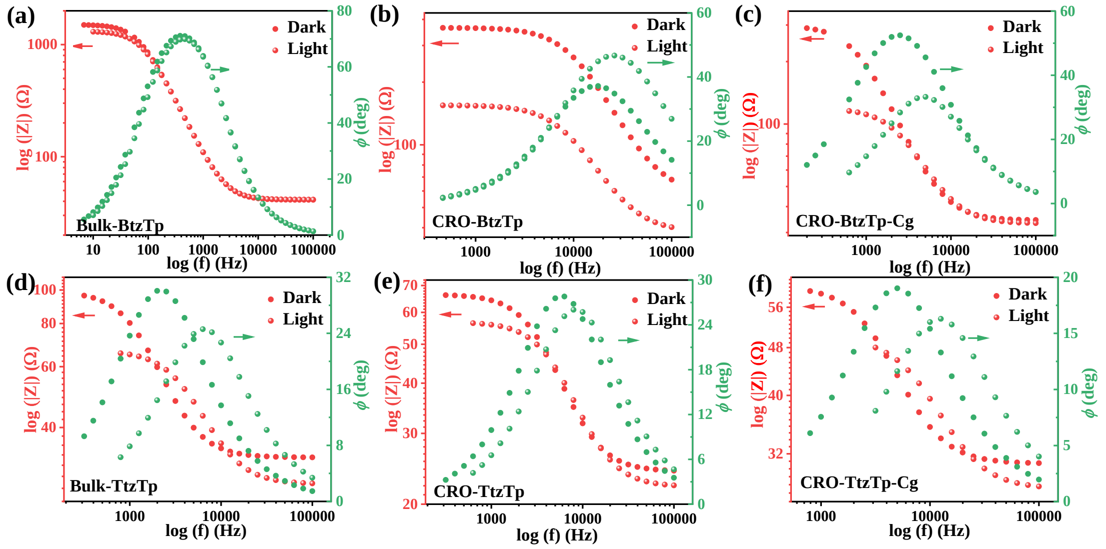

Figure S14. Bode plots of (a) Bulk-BtzTp, CRO-BtzTp, and CRO-BtzTp-Cg; (b) Bulk-TtzTp, CRO-TtzTp, and CRO-TtzTp-Cg.

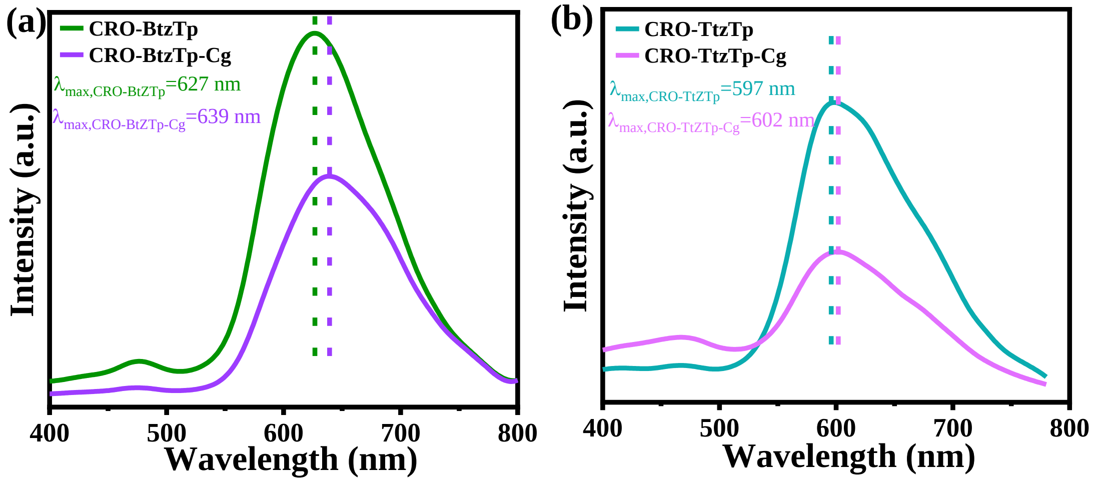

Figure S15. PL spectra of CRO-BtzTp, CRO-BtzTp-Cg, CRO-TtzTp, and CRO-TtzTp-Cg.

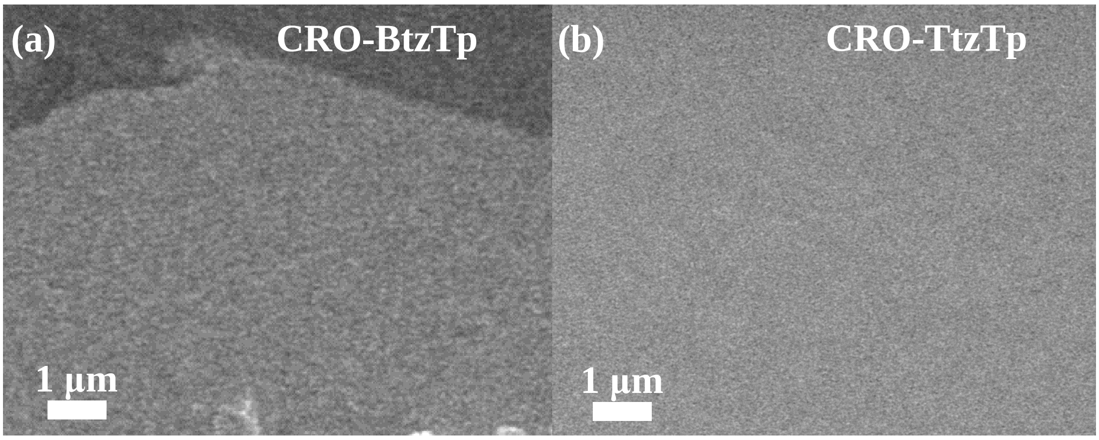

Figure S16. Top−down SEM morphology of spin-coated (a) CRO-BtzTp and (b) CRO-BtzTp.

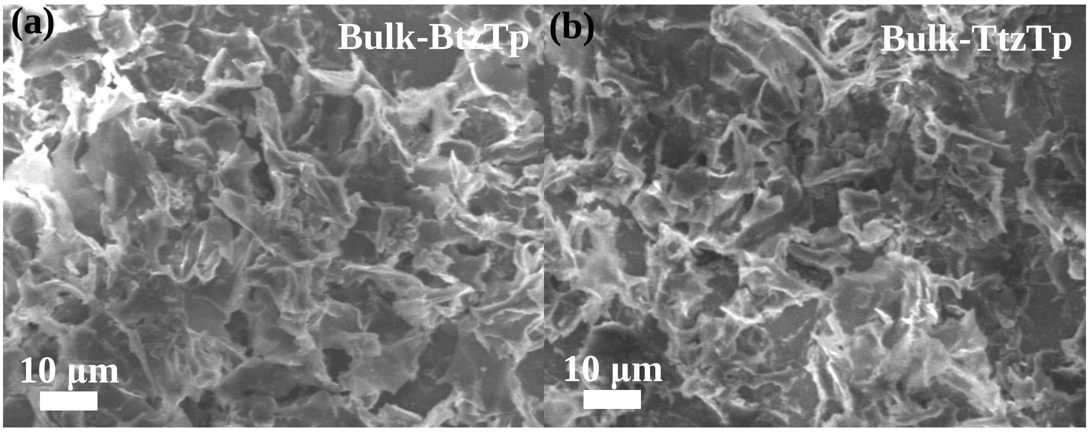

Figure S17. Top−down SEM morphology of spin-coated (a) Bulk-BtzTp and (b) Bulk-TtzTp.

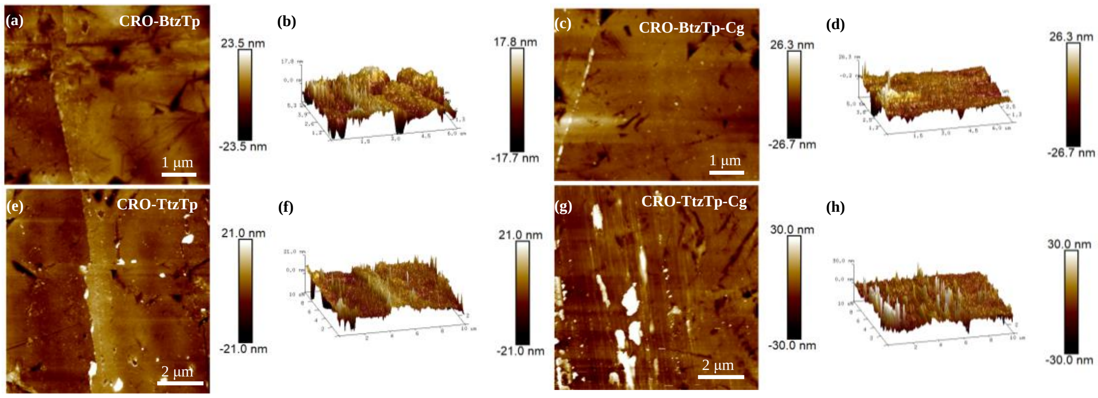

Figure S18. AFM images of CRO-BtzTp, CRO-BtzTp-Cg, CRO-TtzTp, and CRO-TtzTp-Cg.

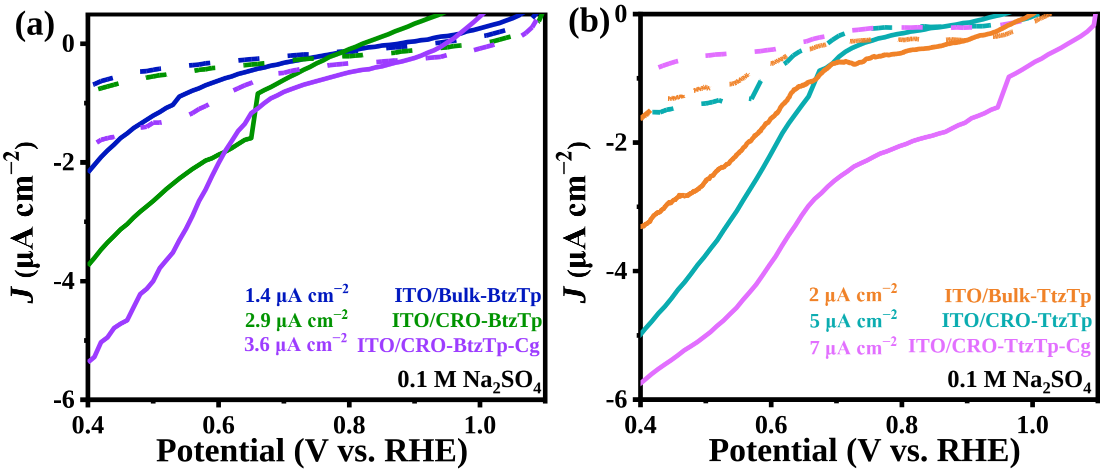

Figure S19. (a, b) LSV curves of ITO/Bulk-BtzTp, ITO/CRO-BtzTp, ITO/CRO-BtzTp-Cg, ITO/Bulk-TtzTp, ITO/CRO-TtzTp, ITO/CRO-TtzTp-Cg.

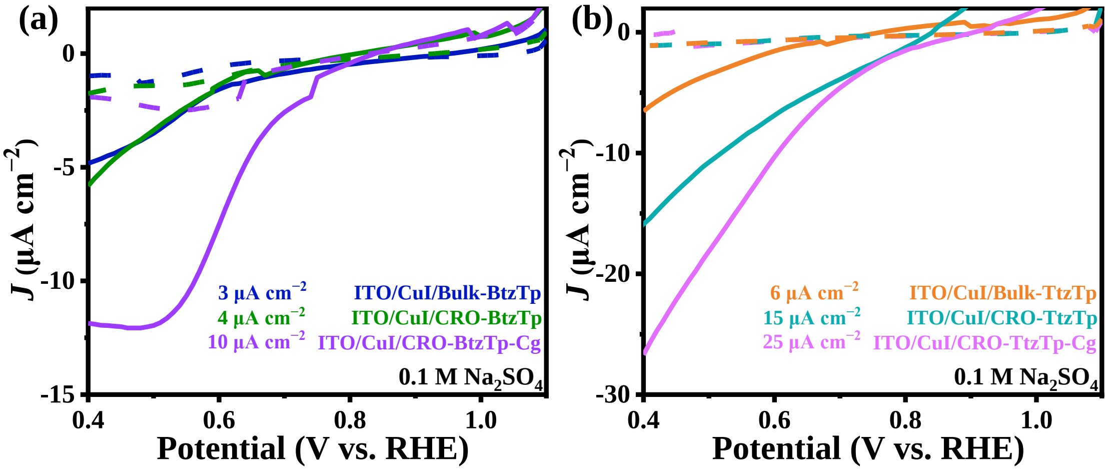

Figure S20. (a, b) LSV curves of ITO/CuI/Bulk-BtzTp, ITO/CuI/CRO-BtzTp, ITO/CuI/CRO-BtzTp-Cg, ITO/CuI/Bulk-TtzTp, ITO/CuI/CRO-TtzTp, ITO/CuI/CRO-TtzTp-Cg.

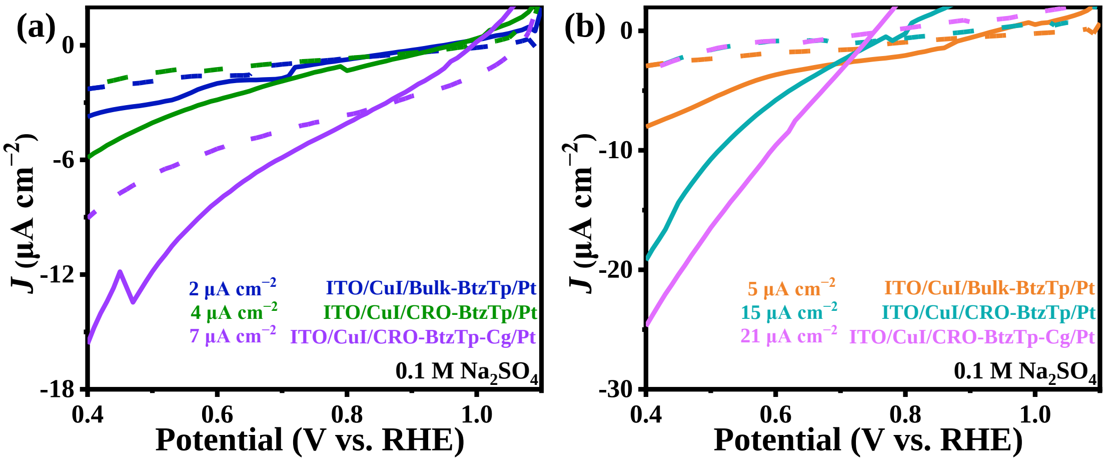

Figure S21. (a, b) LSV curves of ITO/CuI/Bulk-BtzTp/Pt, ITO/CuI/CRO-BtzTp/Pt, ITO/CuI/CRO-BtzTp-Cg/Pt, ITO/CuI/Bulk-TtzTp/Pt, ITO/CuI/CRO-TtzTp/Pt, ITO/CuI/CRO-TtzTp-Cg/Pt.

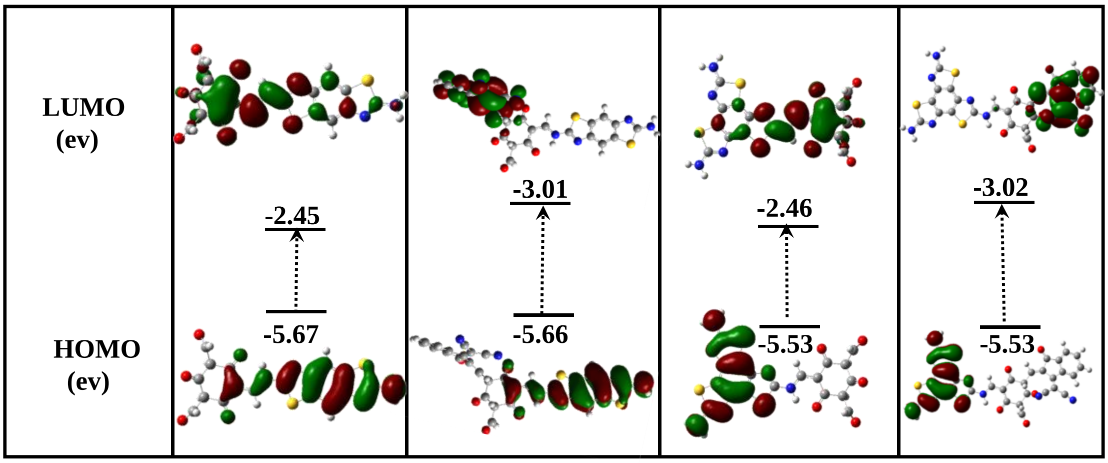

Figure S22. Calculated the HOMO and LUMO.

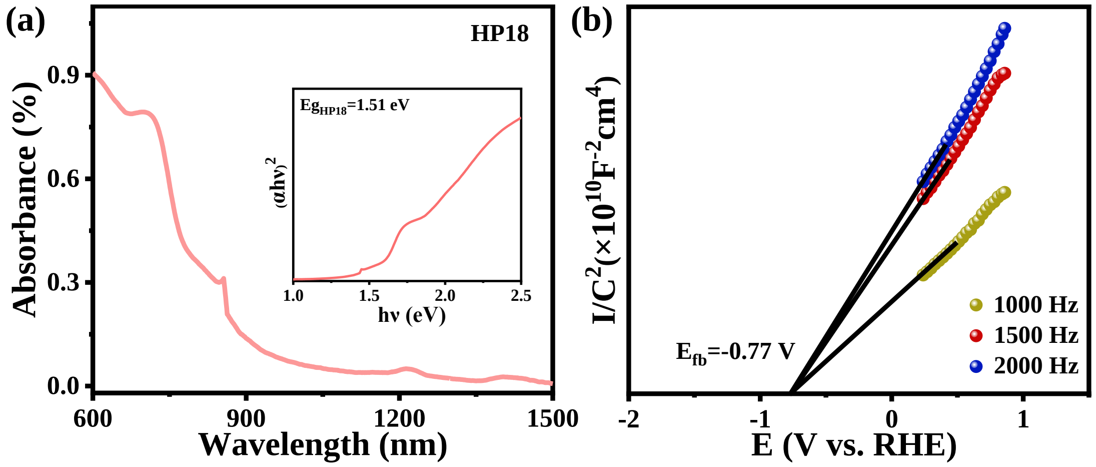

Figure S23. (a) Solid state UV–vis diffuse reflectance spectra of HP18. The inset is Tacu's plot of HP18. (b) Mott–Schottky (M–S) plots

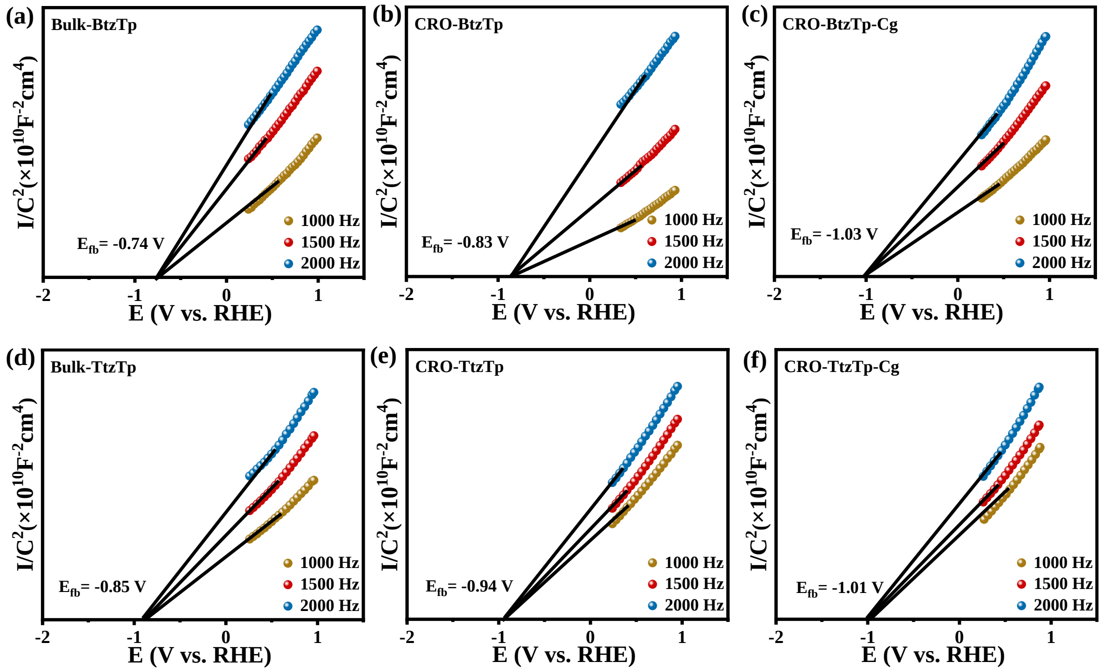

Figure S24. Mott–Schottky (M–S) plots

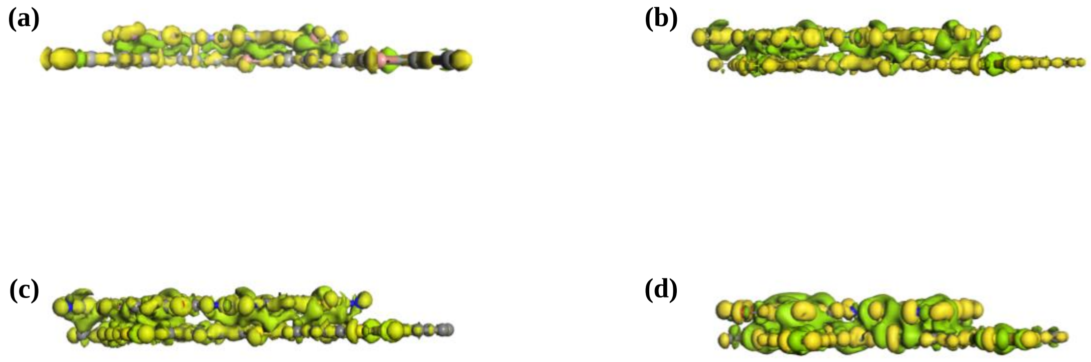

Figure 25. The calculated charge density difference of the (a) HP18:CRO-BtzTp, (b) HP18:CRO-BtzTp-Cg, (c) HP18:CRO-TtzTp, and (d) HP18:CRO-TtzTp-Cg.

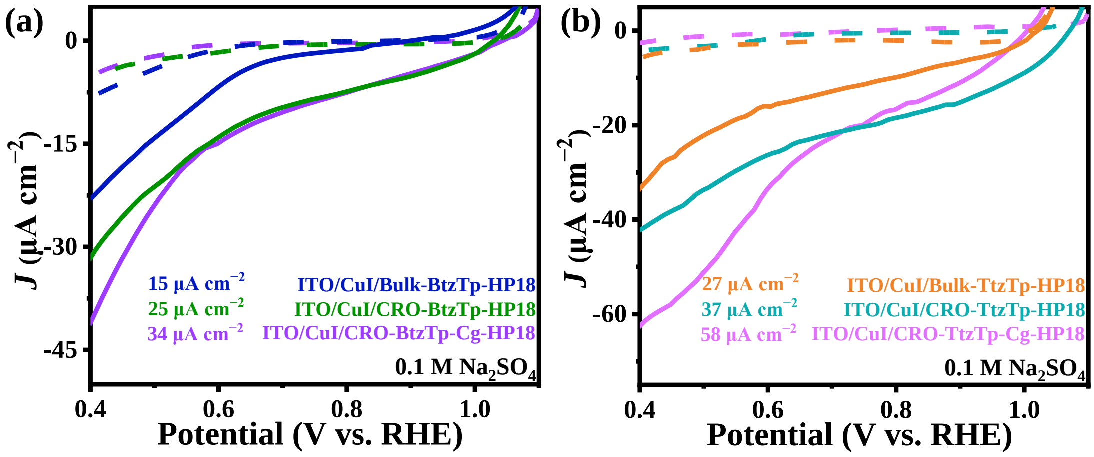

Figure S26. (a, b) LSV curves of ITO/CuI/Bulk-BtzTp-HP18, ITO/CuI/CRO-BtzTp-HP18, ITO/CuI/CRO-BtzTp-Cg-HP18, ITO/CuI/Bulk-TtzTp/SnO_2-HP18, ITO/CuI/CRO-TtzTp-HP18, and ITO/CuI/CRO-TtzTp-Cg-HP18.

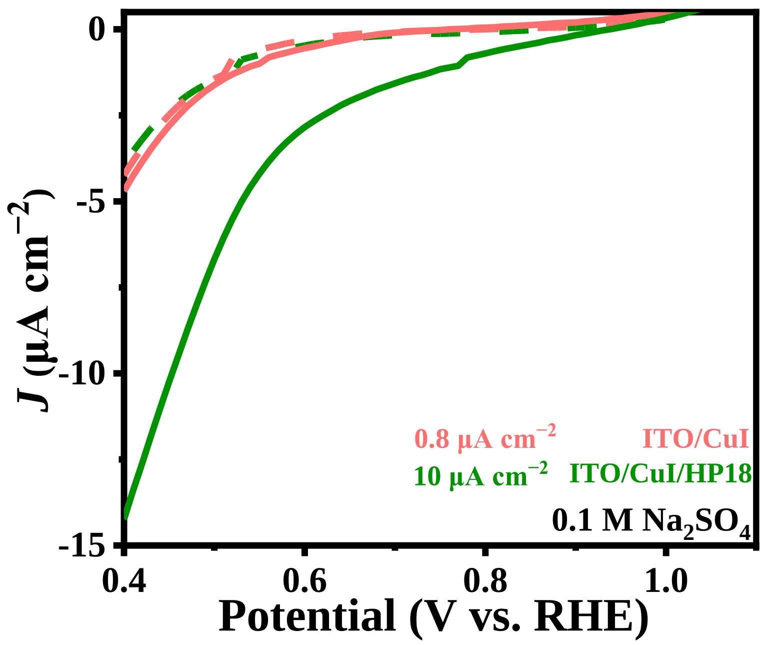

Figure S27. LSV curves of ITO/CuI and ITO/CuI/HP18.

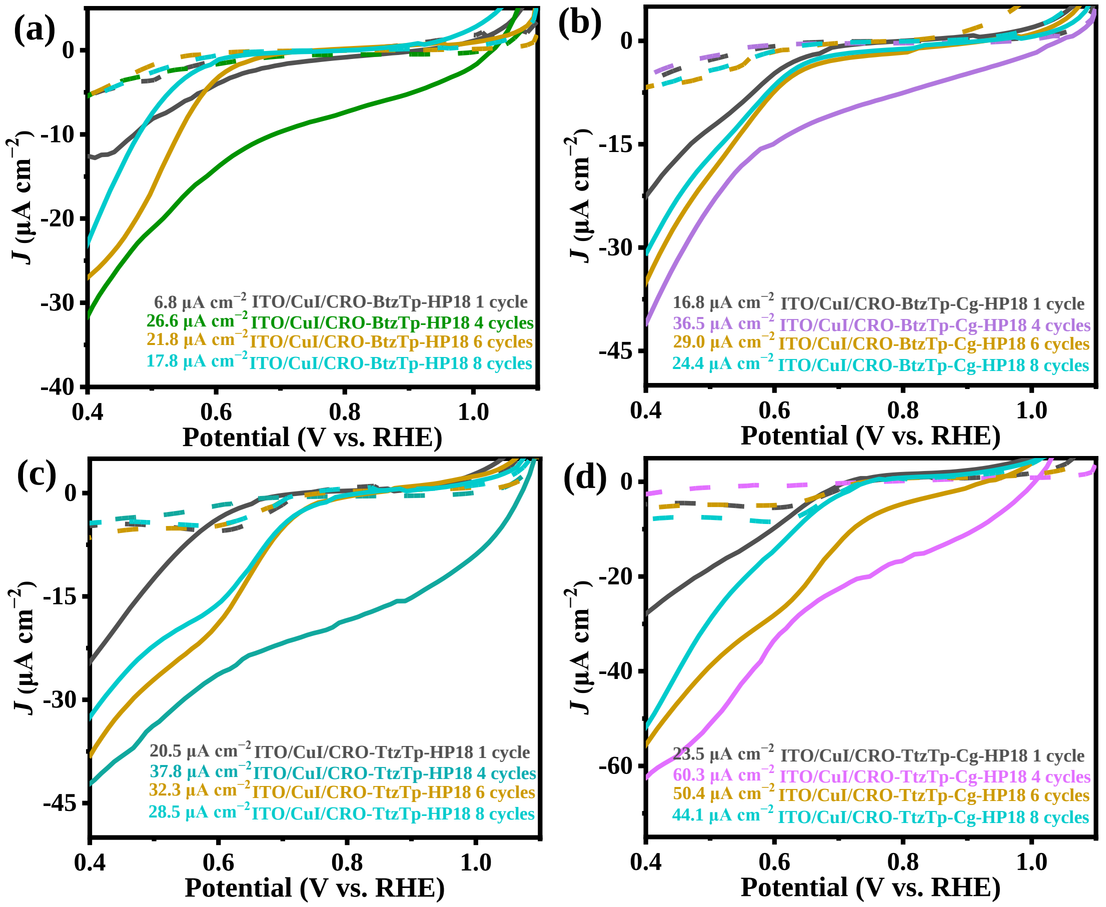

Figure R28. PEC characterization of (a) ITO/CuI/CRO-BtzTp-HP18, (b) ITO/CuI/CRO-BtzTp-Cg-HP18, (c) ITO/CuI/CRO-TtzTp-HP18, and (d) ITO/CuI/CRO-TtzTp-Cg-HP18 photocathodes with different coating cycles.

In the process of fabricating bulk heterojunction devices, we initially optimized the half-device structure by meticulously adjusting the thickness of the active layer. The results reveal that the PEC performance for ITO/CuI/CRO-BtzTp-HP18, ITO/CuI/CRO-BtzTp-Cg-HP18, ITO/CuI/CRO-TtzTp-HP18, and ITO/CuI/CRO-TtzTp-Cg-HP18 is achieved with a four-cycle spin-coating of the active layer. However, beyond the 4-cycle spin-coating, the photocurrent of the photoelectrode for these configurations diminishes. This observation suggests that an increase in film thickness may adversely affect the light absorption capabilities of the photoactive layer.

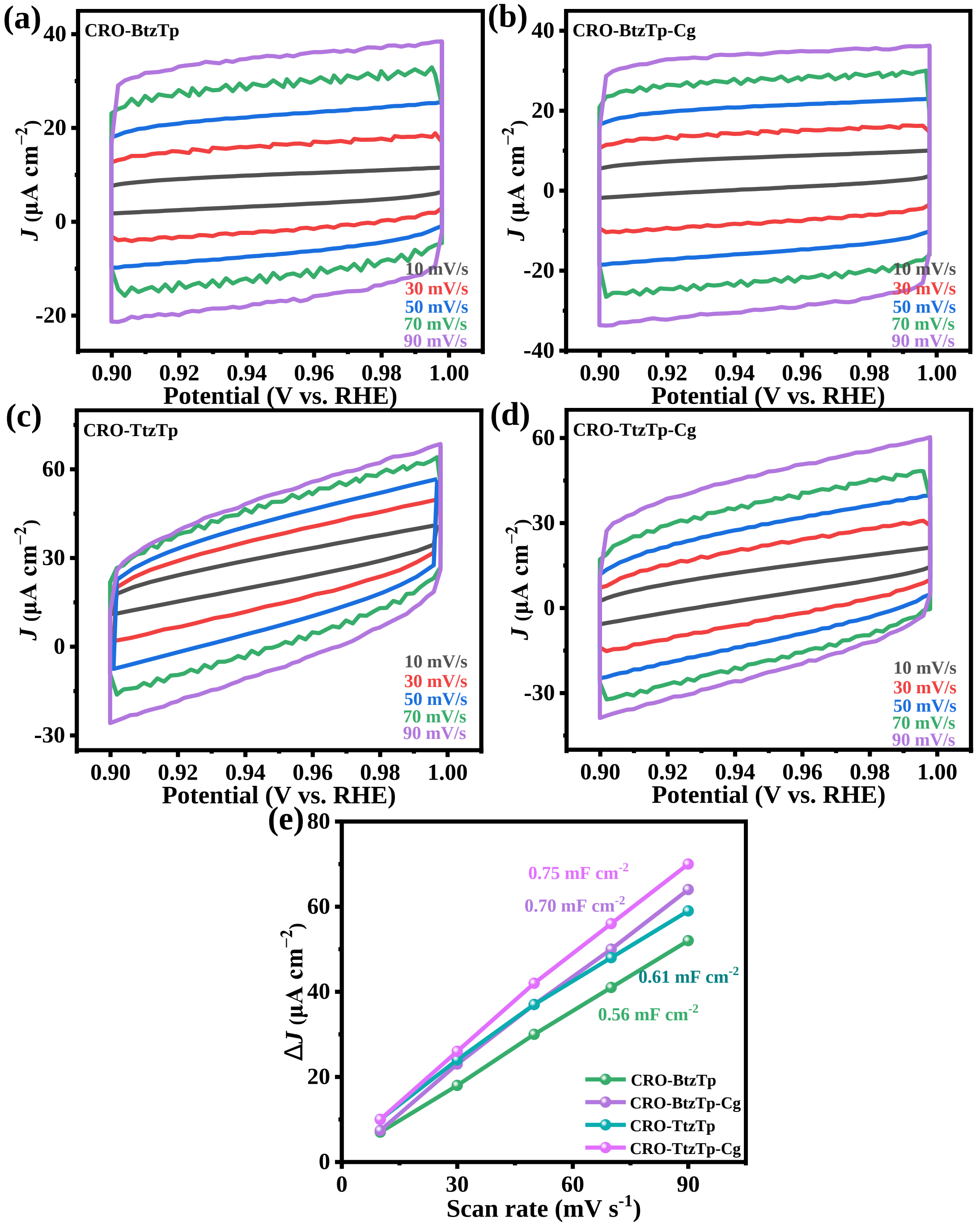

Figure S29. CV curves of (a) ITO/CuI/CRO-BtzTp-HP18/SnO_2/Pt, (b) ITO/CuI/CRO-BtzTp-Cg-HP18/SnO_2/Pt, (c) ITO/CuI/CRO-TtzTp-HP18/SnO_2/Pt and (d) ITO/CuI/CRO-TtzTp-Cg-HP18/SnO_2/Pt photoelectrodes (e) The capacitive current densities at 0.95 V as a function of scan rate for ITO/CuI/CRO-BtzTp-HP18/SnO_2/Pt, ITO/CuI/CRO-BtzTp-Cg-HP18/SnO_2/Pt, ITO/CuI/CRO-TtzTp-HP18/SnO_2/Pt and ITO/CuI/CRO-TtzTp-Cg-HP18/SnO_2/Pt photoelectrodes.

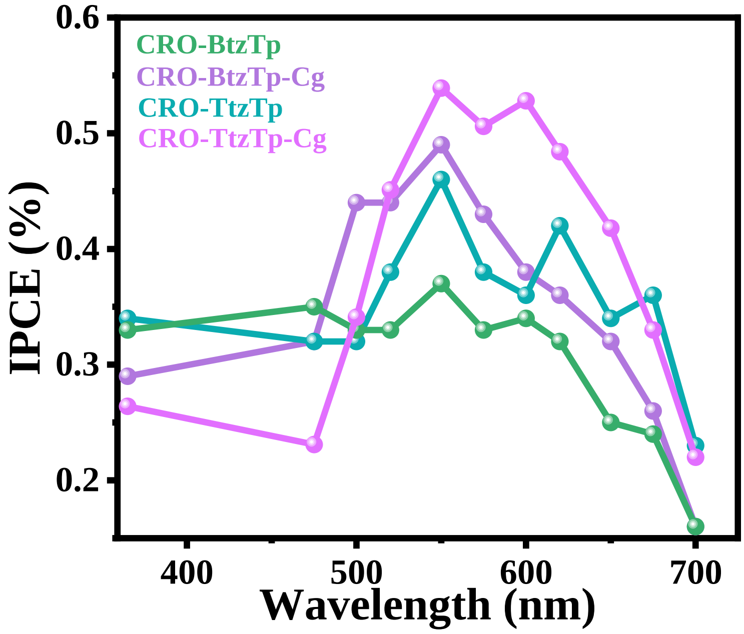

Figure S30. The IPCE spectra of the ITO/CuI/CRO-BtzTp-HP18/SnO_2/Pt, ITO/CuI/CRO-BtzTp-Cg-HP18/SnO_2/Pt, ITO/CuI/CRO-TtzTp-HP18/SnO_2/Pt and ITO/CuI/CRO-TtzTp-Cg-HP18/SnO_2/Pt photoelectrodes. under AM 1.5G irradiation.

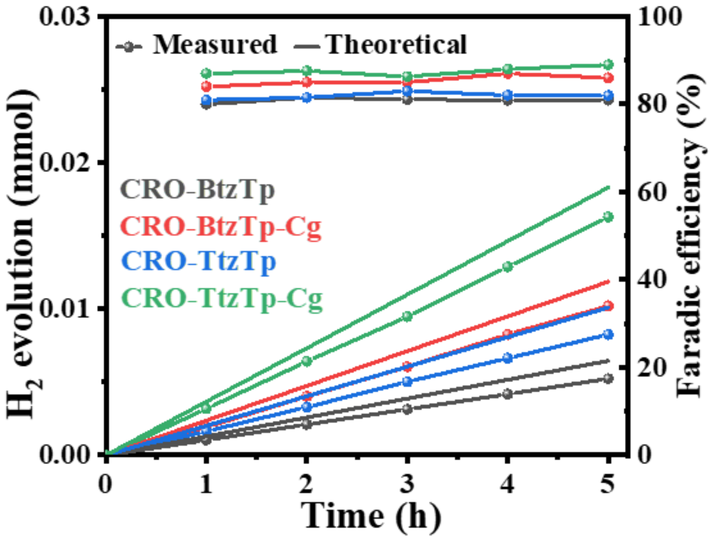

Figure S31. Time courses of H_2 evolution and the recorded Faradaic efficiencies at 1 h time interval.

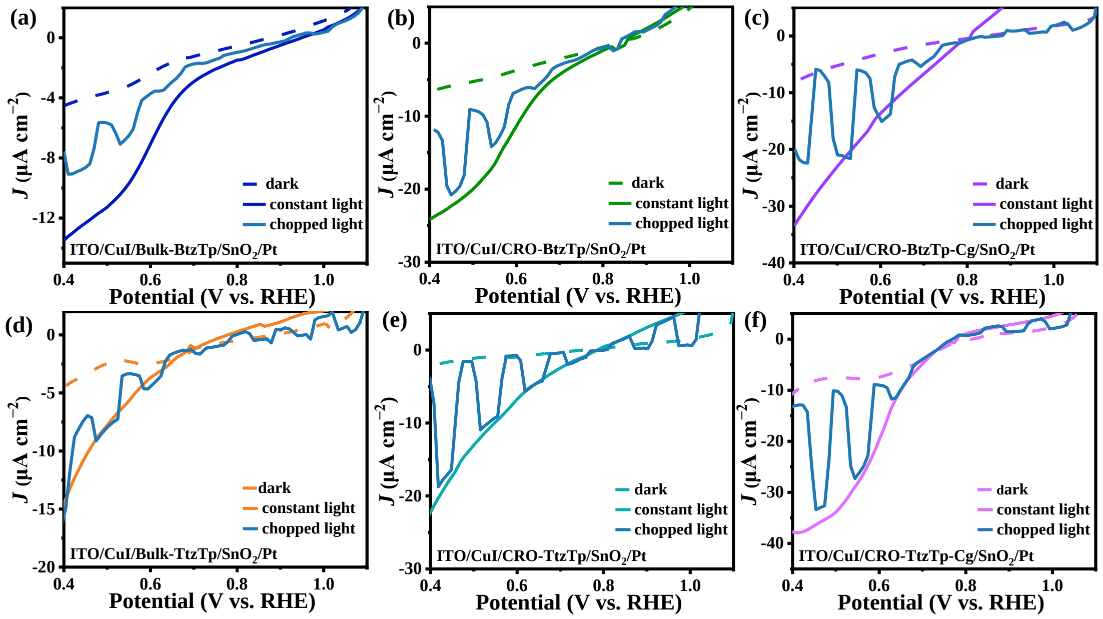

Figure S32. LSV curves of ITO/CuI/Bulk-BtzTp/SnO_2/Pt, ITO/CuI/Bulk-TtzTp/SnO_2/Pt.ITO/CuI/CRO-BtzTp/SnO_2/Pt, ITO/CuI/CRO-BtzTp-Cg/SnO_2/Pt, ITO/CuI/CRO-TtzTp/SnO_2/Pt, and ITO/CuI/CRO-TtzTp-Cg/SnO_2/Pt under constant and chopped light.

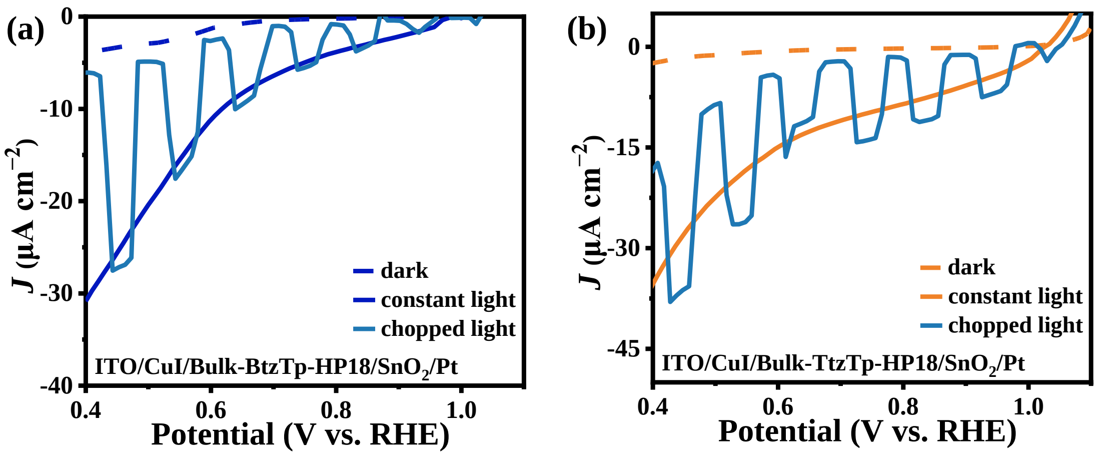

Figure S33. LSV curves of ITO/CuI/Bulk-BtzTp-HP18/SnO_2/Pt (a) and ITO/CuI/Bulk-TtzTp-HP18/SnO_2/Pt (b) under constant and chopped light.

Table S1. The fitted results of EIS data using the equivalent circuit in Figure S13.

Sample	***R*ct (Ω)**	
	**Dark**	**Light**
Bulk-BtzTp	1462	1272
CRO-BtzTp	307	116
CRO-BtzTp-Cg	263	85
Bulk-TtzTp	66	40
CRO-TtzTp	42	34
CRO-TtzTp-Cg	29	22

Table S2. Radiative fluorescence lifetimes and relative percentages of photoexcited charge carriers on CRO-BtzTp and CRO-BtzTp-Cg.

Sample	τ_1/ns–rel.%	τ_2/ns–rel.%	χ^2
CRO-BtzTp	0.50–96.96	0.50–8.64	0.980
CRO-BtzTp-Cg	0.84–67.80	3.55–35.72	0.993

Table S3. Radiative fluorescence lifetimes and relative percentages of photoexcited charge carriers on CRO-TtzTp and CRO-TtzTp-Cg.

**Sample**	**τ_1/μs–rel.%**	**τ_2/μs–rel.%**	**χ^2**
CRO-TtzTp	2.63–82.33	12.36–26.13	0.997
CRO-TtzTp-Cg	3.03–78.83	12.62–27.40	0.998

Table S4. Photocurrent and Δ*J* summary about Btz-based photoelectrodes.

**Btz-based photoelectrodes**	Photocurrent (μA cm^{-2} at 0.4 V *vs.* RHE)	Dark current (μA cm^{-2} at 0.4 V *vs.* RHE)	Δ*J* (μA cm^{-2})
ITO/Bulk-BtzTp	2.2	0.7	1.5
ITO/CRO-BtzTp	3.7	0.8	2.9
ITO/CRO-BtzTp-Cg	5.4	1.8	3.6
ITO/CuI/Bulk-BtzTp	4.8	1.8	3.0
ITO/CuI/CRO-BtzTp	5.7	1.0	4.7
ITO/CuI/CRO-BtzTp-Cg	11.8	1.9	9.9
ITO/CuI/Bulk-BtzTp/Pt	3.8	1.8	2.0
ITO/CuI/CRO-BtzTp/Pt	5.9	2.1	3.8
ITO/CuI/CRO-BtzTp-Cg/Pt	15.6	9.0	6.6
ITO/CuI/Bulk-BtzTp/SnO_2/Pt	13.5	4.4	9.1
ITO/CuI/CRO-BtzTp/SnO_2/Pt	24.2	6.5	17.7
ITO/CuI/CRO-BtzTp-Cg/SnO_2/Pt	33.8	7.9	25.9
ITO/CuI/Bulk-BtzTp-HP18	22.9	7.5	15.4
ITO/CuI/CRO-BtzTp-HP18	31.8	5.2	26.6
ITO/CuI/CRO-BtzTp-Cg-HP18	41.5	5.0	36.5
ITO/CuI/Bulk-BtzTp-HP18/SnO_2/Pt	30.9	3.9	27.0
ITO/CuI/CRO-BtzTp-HP18/SnO_2/Pt	34.6	5.7	28.9
ITO/CuI/CRO-BtzTp-Cg-HP18/SnO_2/Pt	63.6	3.0	60.6

Table S5. Photocurrent and Δ*J* summary about Ttz-based photoelectrodes.

**Ttz-based photoelectrodes**	Photocurrent (μA cm^{-2} at 0.4 V *vs.* RHE)	Dark current (μA cm^{-2} at 0.4 V *vs.* RHE)	Δ*J* (μA cm^{-2})
ITO/Bulk-TtzTp	3.3	1.3	2.0
ITO/CRO-TtzTp	5.0	1.7	3.3
ITO/CRO-TtzTp-Cg	5.7	1.0	4.7
ITO/CuI/Bulk-TtzTp	6.5	0.3	6.2
ITO/CuI/CRO-TtzTp	15.9	1.1	14.8
ITO/CuI/CRO-TtzTp-Cg	26.7	1.1	25.6
ITO/CuI/Bulk-TtzTp/Pt	8.0	2.9	5.1
ITO/CuI/CRO-TtzTp/Pt	19.1	3.4	15.7
ITO/CuI/CRO-TtzTp-Cg/Pt	24.7	3.3	21.4
ITO/CuI/Bulk-TtzTp/SnO_2/Pt	14.9	4.4	10.5
ITO/CuI/CRO-TtzTp/SnO_2/Pt	22.8	2.1	20.7
ITO/CuI/CRO-TtzTp-Cg/SnO_2/Pt	37.8	10.3	27.5
ITO/CuI/Bulk-TtzTp-HP18	33.9	5.5	28.4
ITO/CuI/CRO-TtzTp-HP18	42.1	4.3	37.8
ITO/CuI/CRO-TtzTp-Cg-HP18	62.8	2.5	60.3
ITO/CuI/Bulk-TtzTp-HP18/SnO_2/Pt	36.1	2.4	33.7
ITO/CuI/CRO-TtzTp-HP18/SnO_2/Pt	54.1	10.4	43.7
ITO/CuI/CRO-TtzTp-Cg-HP18/SnO_2/Pt	98.1	3.2	94.9

Section 4. Theoretical calculations

The free energy of the adsorbed state (ΔG) was calculated using the Gaussian 09W program. During geometry and frequency optimization, all atoms were allowed to move freely. Based on the Density Functional Theory (DFT), the quantum cluster calculations were carried out using the B3LYP/6–31G (d, p) basis set. The SMD solvation model was used to consider the solvent (water) effect.

The adsorption energy was calculated according to the following equation:

$$\Delta E_{ad} = E_{CROs-H_{ads}} - E_{CROs} - \frac{1}{2}E_{H_2} \quad \text{(S1)}$$

Where: $E_{\text{CROs-Hads}}$ is the total energy of CROs with absorption of H; E_{CROs} is the energy of the CROs surface; E_{H2} is the energy of hydrogen in the gas phase.

Gibbs free energy was calculated by considering zero-point energy (ZPE) and entropy corrections for the hydrogen evolution reaction, as per the following equation[1]:

$$\Delta G_{\text{H}} = \Delta E_{\text{ad}} + \Delta E_{\text{ZPE}} - T\Delta S \quad \text{(S2)}$$

Where ΔE_{ad} is obtained from equation (1).

In the adsorbed state, hydrogen shows negligible entropy change, due to the vibrational force, so the Gibbs free energy is calculated by considering the following corrections[1]:

$$\Delta G_{\text{H}} = \Delta E_{\text{ad}} + 0.24 \text{ eV} \quad \text{(S3)}$$

B3LYP functional and the 6–31 G (d,p) basis set was employed for the calculations of EA energies (in molecular geometries optimized with B3LYP /6–31 G(d,p)).

TDDFT (time dependent density functional theory) was employed to predict excited state energies and properties to obtain the information of charge transfer.

Reference

[1] X. Li, G. Huang, W. Chen, H. Jiang, S. Qiao, R. Yang, ACS Appl. Mater. Interfaces 2020, 12, 16670–16678.
